# Supplementary material for: Human genetic variation reveals FCRL3 is a lymphocyte receptor for Yersinia pestis
Source: Cell Genom. 2025 Jun 9;5(9):100917. doi: 10.1016/j.xgen.2025.100917 (PMC12534707; doi:10.1016/j.xgen.2025.100917)
Supplement: Document S1. Figures S1–S3 and Tables S2–S4 [file mmc1.pdf]

Cell Genomics, Volume *5*

Supplemental information

Human genetic variation reveals FCRL3 is a lymphocyte receptor for *Yersinia pestis*

Rachel M. Keener, Sam Shi, Trisha Dalapati, Liuyang Wang, Nicolás M. Reinoso-Vizcaino, Micah A. Luftig, Samuel I. Miller, Timothy J. Wilson, and Dennis C. Ko

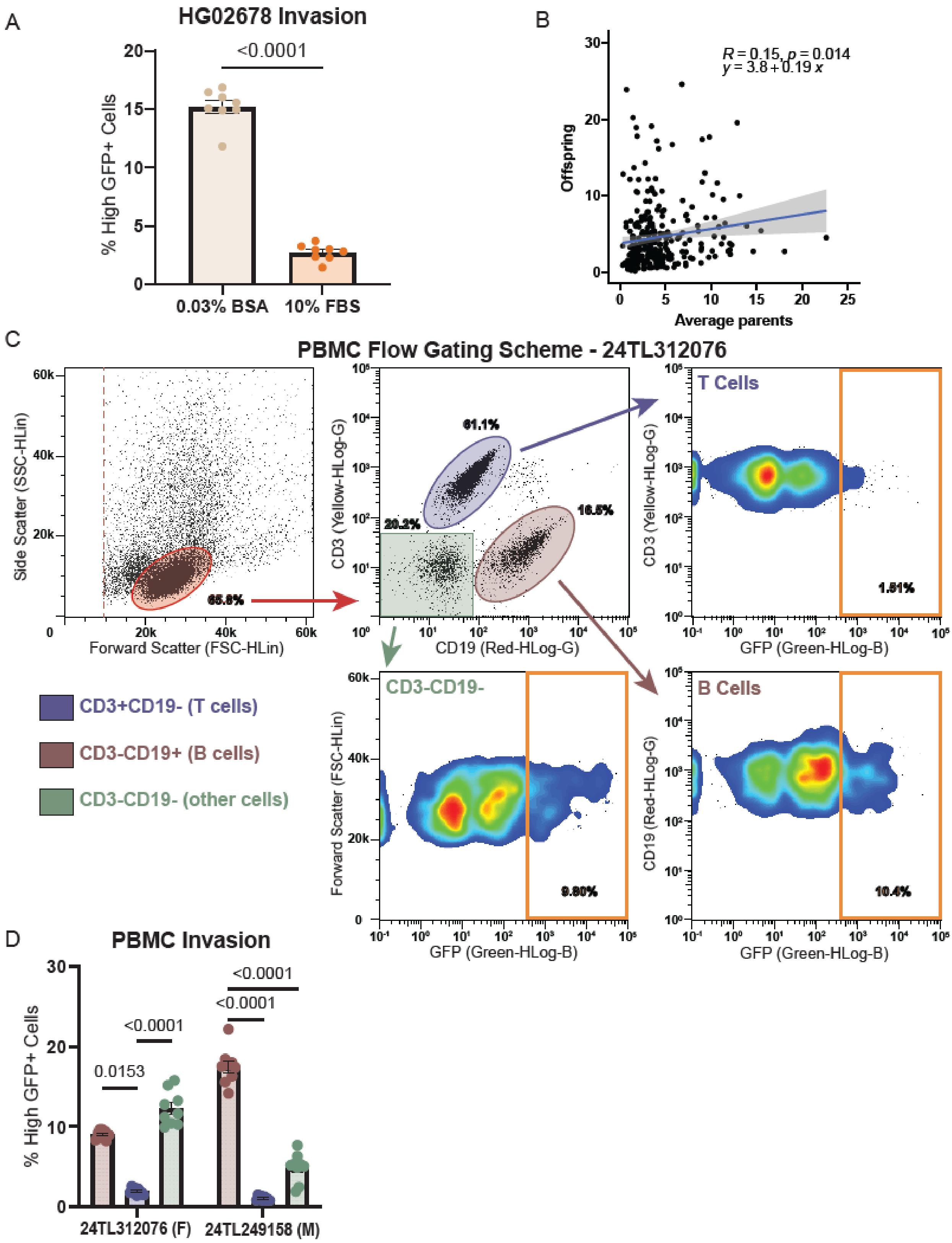
A
HG02678 Invasion
<0.0001
% High GFP+ Cells
0.03% BSA
10% FBS
B
R = 0.15, p = 0.014
y = 3.8 + 0.19 x
Offspring
Average parents
C
PBMC Flow Gating Scheme - 24TL312076
Side Scatter (SSC-HLin)
Forward Scatter (FSC-HLin)
CD3 (Yellow-HLog-G)
CD19 (Red-HLog-G)
61.1%
16.5%
20.2%
T Cells
1.51%
GFP (Green-HLog-B)
CD3-CD19-
9.80%
B Cells
10.4%
CD19 (Red-HLog-G)
CD3+CD19- (T cells)
CD3-CD19+ (B cells)
CD3-CD19- (other cells)
D
PBMC Invasion
% High GFP+ Cells
0.0153
<0.0001
<0.0001
<0.0001
24TL312076 (F)
24TL249158 (M)

Figure S1. *Y. pestis* invasion into LCLs and PBMCs, related to figure 1. (**A**) Flow cytometric measurement of *Y. pestis* (KIM6+ +p67GFP3.1) invasion of LCLs at 4 hpi in RPMI media with either 10% FBS or 0.03% BSA after gentamicin protection assay. 2 experiments are grand mean normalized with 8 total replicates plotted for each condition. An unpaired t-test was performed to determine significance. (**B**) Parent-offspring linear regression of *Y. pestis* invasion into LCLs gives a correlation coefficient of 0.15, a slope and estimated heritability of 0.1883 and a p-value = 0.014. (**C**) Flow cytometry gating scheme for *Y. pestis* infected PBMCs. After infecting cells in serum free RPMI +0.03% BSA for 1 hour, a gentamicin protection assay was performed. Cells were then stained with CD19 (B cell marker) and CD3 (T cell marker) before counting 15000 cells on a Guava Easycyte HT flow cytometer using the InCyte program. First, viable cells are gated in the red gate in the top left dot plot. T cells are then defined in the blue gate, B cells in brown, and CD3-CD19- cells in green in the top middle dot plot. Each population is then separated, and invasion (GFP^{HIGH}) is measured in three density plots. (**D**) Bar graph quantifying flow cytometry of PBMCs demonstrates B cell preference for invasion. Dots represent 3 replicates in each of 3 experiments for PBMCs from each donor. P-value determined by two-way ANOVA with Sidak’s multiple comparison test.

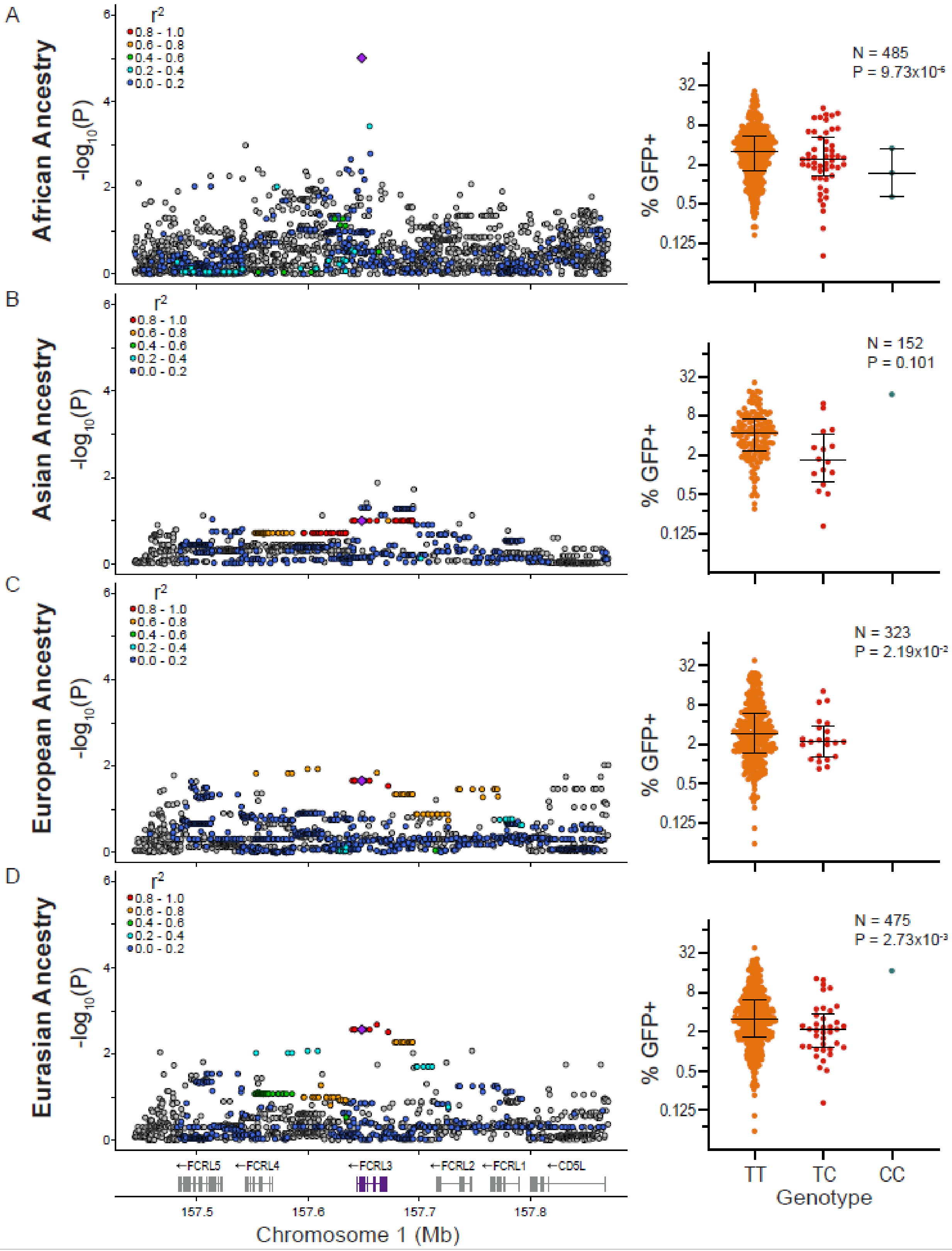
A
B
C
D
African Ancestry
Asian Ancestry
European Ancestry
Eurasian Ancestry
-log10(P)
r2
0.8 - 1.0
0.6 - 0.8
0.4 - 0.6
0.2 - 0.4
0.0 - 0.2
N = 485
P = 9.73x10-6
N = 152
P = 0.101
N = 323
P = 2.19x10-2
N = 475
P = 2.73x10-3
% GFP+
←FCRL5
←FCRL4
←FCRL3
←FCRL2
←FCRL1
←CD5L
157.5
157.6
157.7
157.8
Chromosome 1 (Mb)
TT
TC
CC
Genotype

Figure S2. **Locus zoom and genotypic mean plots of LCLs stratified by ancestry, related to figure 1.** Local Manhattan plots and genotypic mean plots stratified by African (GWD, YRI, ESN), Asian (JPT, CHB, KHV), European (CEU, IBS), or EurAsian (JPT, CHB, KHV, CEU, IBS) ancestries. Plot shows the *FCRL3* locus flanked on either side by 200kb. A purple diamond denotes rs2282284 and LD with SNPs in the locus is shown by red >= 0.8, orange = 0.6-0.8, green = 0.4-0.6, light blue = 0.2-0.4, dark blue < 0.2, and grey has no LD data. LD was determined by all populations within the stratified group. $-\log_{10}(p)$ values at the rs2282284 locus was plotted and phenotypes were plotted by genotype. All populations demonstrate the C allele of rs2282284 is associated with decreased invasion.

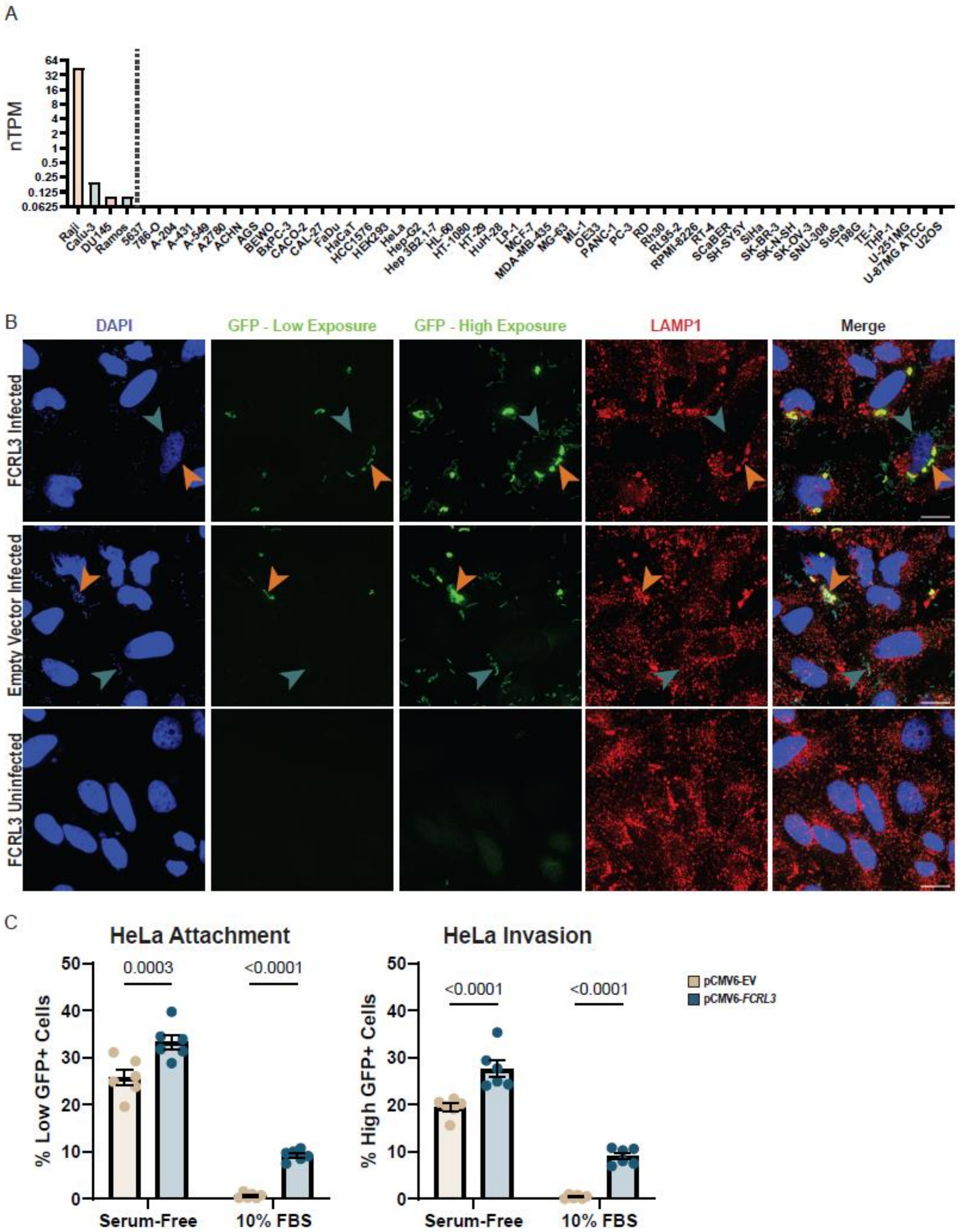
A
nTPM
64
32
16
8
4
2
1
0.5
0.25
0.125
0.0625
Raji
Calu-3
DU145
Ramos
5637
786-O
A-204
A-431
A-549
A2780
ACHN
AGS
BEWO
BxPC-3
CACO-2
CAL-27
FaDu
HaCaT
HCC1576
HEK293
HeLa
Hep-G2
Hep 3B2.1-7
HL-60
HT-1080
HT-29
HuH-28
LP-1
MCF-7
MDA-MB-435
MG-63
ML-1
OE33
PANC-1
PC-3
RD
Rh30
RL95-2
RPMI-8226
RT-4
SCaBER
SH-SY5Y
SiHa
SK-BR-3
SK-N-SH
SK-OV-3
SNU-308
SuSa
T98G
TE-1
THP-1
U-251MG
U-87MG ATCC
U2OS
B
DAPI
GFP - Low Exposure
GFP - High Exposure
LAMP1
Merge
FCRL3 Infected
Empty Vector Infected
FCRL3 Uninfected
C
HeLa Attachment
% Low GFP+ Cells
0.0003
<0.0001
Serum-Free
10% FBS
HeLa Invasion
% High GFP+ Cells
<0.0001
<0.0001
Serum-Free
10% FBS
50
40
30
20
10
0
pCMV6-EV
pCMV6-FCRL3

Figure S3. No FCRL3 protein is detected in HeLa cells and intracellular *Y. pestis* is in LAMP+ vesicles at 4 hpi, related to figure 2. (**A**) Bar graphs of expression in common cell lines from the Human Protein Atlas[S1] show expression only in lymphoma, Calu-3, and DU145 cell lines and no expression in HeLa. (**B**) GFP^{high} cells have intracellular *Y. pestis* within LAMP1+ vacuoles. HeLa cells transfected with empty vector or *FCRL3* plasmid were infected with KIM6+ +p67GFP3.1 *Y. pestis* for 1 hr, treated with gentamicin for 1 hr, and induced with IPTG for 2 hr prior to fixation with 4% paraformaldehyde. After incubating for 30 minutes in block/perm, DNA was stained with 2.5µM DAPI, LAMP1 was stained red with a 1:20 dilution of H4A3-s mouse LAMP1 antibody (Developmental Studies Hybridoma Bank), and bacteria are shown in green at high exposure to include the GFP^{low} population. GFP^{low} bacteria are indicated with a teal arrowhead and GFP^{high} bacteria with an orange arrowhead. Images were taken on a Zeiss Observer Z1 inverted microscope with a 63x water objective. A 20µm scale bar is located on the merged image. ImageJ was used to adjust and crop images and to add scale bars. (**C**) Serum reduces FCRL3-independent attachment and invasion in HeLa cells overexpressing pCMV6 empty vector or FCRL3. HeLa cells were transfected with empty vector or FCRL3 plasmid and assayed for attachment and invasion + or – 10% Fetal Bovine Serum by flow cytometry. Three biological replicates in each of two experiments were plotted and a two-way ANOVA was performed with Uncorrected Fisher's LSD multiple comparison test to determine the P-value. Experiments were normalized by grand mean.

Table S2. sgRNAs and primers for CRISPR mutagenesis and validation related to figure 2 and STAR methods.

GENE	GUIDE 1	GUIDE 2	GUIDE 3	FWD primer	REV primer
CD46	GAGAAACAUGUCCAUAUAUA	AACUCGUAAGUCCCAUUUGC	UUGCUCCUUAGAGGAAAUAA	TGCCTGGGTGAATATGAATCTT	TGTCAGAAACAGCAAGTAGTTTTG
FCRL3	AAUUUCCAGGCUCUGUAAUU	GAUACCAAUAUGUGUCUCCC	CUGUGGACCAUGGAGGAUUG	TCTGCCTAGGATCCCTGCAT	ACCCTGGTCCTGACTGGA

Table S3. GeneBlocks used in IG-like domain deletion and insertion cloning, related to figure 5 and STAR methods.

FCRL3 Ig-Like Domain	Parental Plasmid	Sequence
D1 Deletion	pCMV6 FCRL3	AGCTATGCGATCGCCATGCTTCTGTGGCTGCTGCTGCTGATCCTGACTCCTGGAAGAGAACAATCAGGGGTGGCCCCTGACTGGCTGATCCTGCAGGCTTTACATCCTGTCTTTGAAGGAGACAATGTCATTCTGAGATGTCAGGGGAAAGACAACAAAAACACTCATCAAAAGGTTTACTACAAGGATGGAAAACAGCTTCCTAATAGTTATAATTTAGAGAAGATCACAGTGAATTCAGTCTCCAGGGATAATAGCAAATATCATTGTACTGCTTATAGGAAGTTTTACATACTTGACATTGAAGTAACTTCAAAACCCCTAAATATCCAAGTTCAAGAGCTGTTTCTACATCCTGTGCTGAGAGCCAGCTCTTCCACGCCCATAGAGGGGAGTCCCATGACCCTGACCTGTGAGACCCAGCTCTCTCCACAGAGGCCAGATGTCCAGCTGCAATTCTCCCTCTTCAGAGATAGCCAGACCCTCGGATTGGGCTGGAGCAGGTCCCCCAGACTCCAGATCCCTGCCATGTGGACTGAAGACTCAGGGTCTTACTGGTGTGAGGTGGAGACAGTGACTCACAGCATCAAAAAAAGGAGCCTGAGATCTCAGATACGTGTACAGAGAGTCCCTGTGTCTAATGTGAATCTAGATCAGCC
D2 Deletion	pCMV6 FCRL3	AGCTATGCGATCGCCATGCTTCTGTGGCTGCTGCTGCTGATCCTGACTCCTGGAAGAGAACAATCAGGGGTGGCCCCAAAAGCTGTACTTCTCCTCAATCCTCCATGGTCCACAGCCTTCAAAGGAGAAAAAGTGGCTCTCATATGCAGCAGCATATCACATTCCCTAGCCCAGGGAGACACATATTGGTATCACGATGAGAAGTTGTTGAAAATAAAACATGACAAGATCCAAATTACAGAGCCTGGAAATTACCAATGTAAGACCCGAGGATCCTCCCTCAGTGATGCCGTGCATGTGGAATTTTCAATCCAAGTTCAAGAGCTGTTTCTACATCCTGTGCTGAGAGCCAGCTCTTCCACGCCCATAGAGGGGAGTCCCATGACCCTGACCTGTGAGACCCAGCTCTCTCCACAGAGGCCAGATGTCCAGCTGCAATTCTCCCTCTTCAGAGATAGCCAGACCCTCGGATTGGGCTGGAGCAGGTCCCCCAGACTCCAGATCCCTGCCATGTGGACTGAAGACTCAGGGTCTTACTGGTGTGAGGTGGAGACAGTGACTCACAGCATCAAAAAAAGGAGCCTGAGATCTCAGATACGTGTACAGAGAGTCCCTGTGTCTAATGTGAATCTAGATCAGCC
D3 Deletion	pCMV6 FCRL3	AGCTATGCGATCGCCATGCTTCTGTGGCTGCTGCTGCTGATCCTGACTCCTGGAAGAGAACAATCAGGGGTGGCCCCAAAAGCTGTACTTCTCCTCAATCCTCCATGGTCCACAGCCTTCAAAGGAGAAAAAGTGGCTCTCATATGCAGCAGCATATCACATTCCCTAGCCCAGGGAGACACATATTGGTATCACGATGAGAAGTTGTTGAAAATAAAACATGACAAGATCCAAATTACAGAGCCTGGAAATTACCAATGTAAGACCCGAGGATCCTCCCTCAGTGATGCCGTGCATGTGGAATTTTCACCTGACTGGCTGATCCTGCAGGCTTTACATCCTGTCTTTGAAGGAGACAATGTCATTCTGAGATGTCAGGGGAAAGACAACAAAAACACTCATCAAAAGGTTTACTACAAGGATGGAAAACAGCTTCCTAATAGTTATAATTTAGAGAAGATCACAGTGAATTCAGTCTCCAGGGATAATAGCAAATATCATTGTACTGCTTATAGGAAGTTTTACATACTTGACATTGAAGTAACTTCAAAACCCCTAAATATCCAAGTTCAAGAGCTGTTTCTACATAAAAGGAGCCTGAGATCTCAGATACGTGTACAGAGAGTCCCTGTGTCTAATGTGAATCTAGATCAGCC

D1 Insertion	pFLAG-CMV-3<br>FCRL1	AGCTATAAGCTTGCGGCCCCAAAAGCTGTACTTCTCCTCAATCCTCCATGGT<br>CCACAGCCTTCAAAGGAGAAAAAGTGGCTCTCATATGCAGCAGCATATCAC<br>ATTCCCTAGCCCAGGGAGACACATATTGGTATCACGATGAGAAGTTGTTGA<br>AAATAAAACATGACAAGATCCAAATTACAGAGCCTGGAAATTACCAATGTA<br>AGACCCGAGGATCCTCCCTCAGTGATGCCGTGCATGTGGAATTTTCAGCGG<br>AGCTGTTTTTGATAGCCAGCCCCTCCCATCCCACAGAGGGGAGCCCAGTGA<br>CCCTGACGTGTTCAGCC

Table S4. Oligonucleotides for Quikchange mutagenesis of FCRL3, related to figure 5 and STAR Methods.

mutation	Forward	reverse
N721S	catgaagaagatgatgaagaaagctatgagaatgtaccacgtgta	tacacgtggtacattctcatagctttcttcatcatcttcttcatg
Y650F	ggagctggagccaatgttcagcaatgtaaatcctg	caggatttacattgctgaacattggctccagctcc
Y662F	ctggagatagcaacccgatttttcccagatctg	cagatctgggaaaaatcgggttgctatctccag
Y692F	gaggaacttacagtcctcttttcagaactgaagaagaca	tgtcttcttcagttctgaaaagaggactgtaagttcctc
Y722F	cccatgaagaagatgatgaagaaaactttgagaatgtaccac	gtggtacattctcaaagttttcttcatcatcttcttcatggg

Supplemental references

S1. Uhlen, M., Fagerberg, L., Hallstrom, B.M., Lindskog, C., Oksvold, P., Mardinoglu, A., Sivertsson, A., Kampf, C., Sjostedt, E., Asplund, A., et al. (2015). Proteomics. Tissue-based map of the human proteome. Science *347*, 1260419. 10.1126/science.1260419.
