# Supplementary material for: Human genetic variation reveals FCRL3 is a lymphocyte receptor for Yersinia pestis
Source: Cell Genom. 2025 Jun 9;5(9):100917. doi: 10.1016/j.xgen.2025.100917 (PMC12534707; doi:10.1016/j.xgen.2025.100917)
Supplement: Document S2. Article plus supplemental information [file mmc3.pdf]

Cell Genomics

Article

Human genetic variation reveals FCRL3 is a lymphocyte receptor for *Yersinia pestis*

Graphical abstract

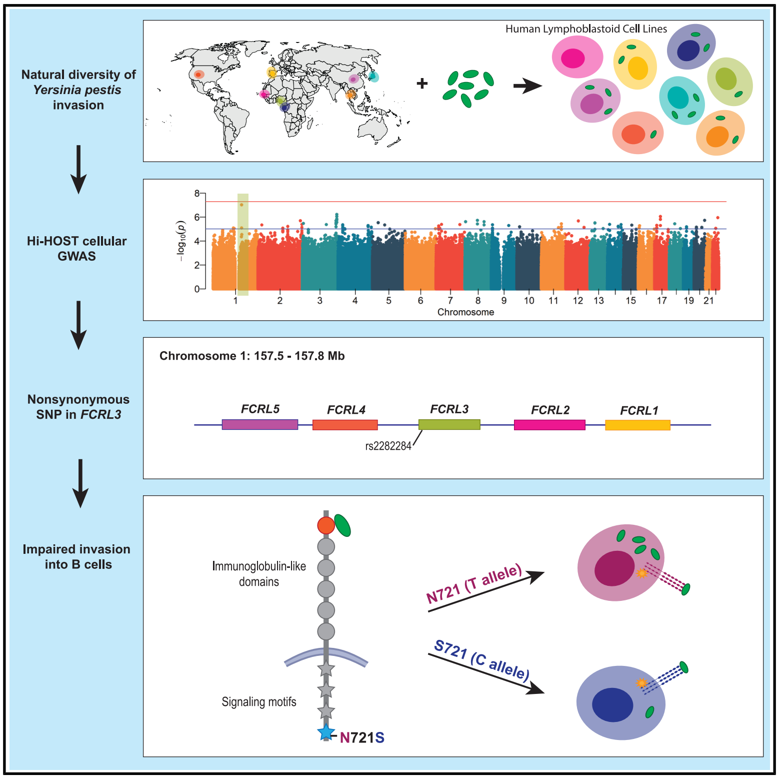

Authors

Rachel M. Keener, Sam Shi, Trisha Dalapati, ..., Samuel I. Miller, Timothy J. Wilson, Dennis C. Ko

Correspondence

dennis.ko@duke.edu

In brief

Keener et al. conducted a cellular GWAS of nearly 1,000 lymphoblastoid cell lines to identify a common missense variant affecting FCRL3 (Fc receptor-like 3) as an important factor regulating *Yersinia pestis* invasion into host cells. FCRL proteins directly bind *Y. pestis* and cluster at sites of attachment.

Highlights

- Cellular GWAS revealed FCRL3 N721S is associated with *Yersinia pestis* invasion
- FCRL3 is a cell surface protein that clusters at sites of *Y. pestis* attachment
- FCRL protein redundancy revealed molecular features for direct binding and invasion
- The same genetic variant is associated with the risk of chronic hepatitis C

Keener et al., 2025, Cell Genomics *5*, 100917
September 10, 2025 © 2025 The Authors. Published by Elsevier Inc.
https://doi.org/10.1016/j.xgen.2025.100917

CellPress

Cell Genomics

CellPress
OPEN ACCESS

Article

Human genetic variation reveals FCRL3 is a lymphocyte receptor for *Yersinia pestis*

Rachel M. Keener,[1,2] Sam Shi,[1] Trisha Dalapati,[1] Liuyang Wang,[1] Nicolás M. Reinoso-Vizcaino,[1] Micah A. Luftig,[1] Samuel I. Miller,[3] Timothy J. Wilson,[4] and Dennis C. Ko[1,2,5,6,*]
[1]Department of Molecular Genetics and Microbiology, School of Medicine, Duke University, Durham, NC 27710, USA
[2]University Program in Genetics and Genomics, Duke University, Durham, NC 27710, USA
[3]Departments of Genome Sciences, Medicine, and Microbiology, University of Washington, Seattle, WA 98195, USA
[4]Department of Microbiology, Miami University, Oxford, OH 45056, USA
[5]Division of Infectious Diseases, Department of Medicine, School of Medicine, Duke University, Durham, NC 27710, USA
[6]Lead contact
*Correspondence: dennis.ko@duke.edu
https://doi.org/10.1016/j.xgen.2025.100917

SUMMARY

Yersinia pestis is the bacterium responsible for plague, one of the deadliest diseases in history. To discover human genetic determinants of *Y. pestis* infection, we utilized nearly 1,000 genetically diverse lymphoblastoid cell lines in a cellular genome-wide association study. A nonsynonymous SNP, rs2282284 (N721S), in *Fc receptor-like 3* (*FCRL3*) was associated with bacterial invasion of host cells ($p = 9 \times 10^{-8}$). Overexpressed FCRL3 facilitated attachment and invasion of *Y. pestis* and colocalized with *Y. pestis* at attachment sites. These properties were variably conserved across the FCRL family, revealing an immunoglobulin-like domain and signaling motifs shared by FCRL3 and FCRL5 to be necessary for attachment and invasion. Direct binding to FCRL5 extracellular domain was confirmed, and B cells (the primary cells that express FCRLs) were preferentially invaded by *Y. pestis*. Thus, *Y. pestis* hijacks FCRL proteins, possibly taking advantage of an immune receptor to create a lymphocyte niche during infection.

INTRODUCTION

Pandemics have been potent evolutionary forces throughout human history, selecting for host genetic resistance alleles that likely still impact infectious disease.[1,2] *Yersinia pestis*, the causative agent of the disease state plague, was responsible for the deadliest pandemic in history, the Black Death of the 14th century.[3] Left untreated, the bubonic plague mortality varied from 30% to 50% historically,[4] while the septicemic and pneumonic forms of disease caused almost 100% fatality.[5] Candidate gene studies,[6,7] genome-wide cellular screens,[8–10] and ancient DNA studies[10,11] have implicated various host genes in *Y. pestis* infection. However, a genome-wide association study (GWAS) of *Y. pestis* infection has never been reported.

Genetic association studies have been critical in revealing the genetic architecture of human susceptibility to infectious disease and have also identified host factors that are important in regulating susceptibility and severity of infection.[12,13] Perhaps the most important host factors in controlling susceptibility to infection are surface receptors that mediate attachment and entry of pathogens. In this regard, human genetic differences affecting entry receptors have been found to confer near-complete resistance against two viral infections. Most famously, *CCR5 Δ32* (rs333) is a naturally occurring allele in an HIV-1 co-receptor that protects against HIV-1 infection.[14–16] Indeed, the first three individuals who have been cured of HIV-1 infection received stem cell transplants from homozygous *CCR5 Δ32* donors.[17,18] Similarly, a nonsense mutation in *FUT2* (rs601338) results in the absence of specific fucosylated oligosaccharides and a loss of susceptibility to infection with certain Norwalk virus strains that use the sugars to attach to host cells.[19] Thus, there is strong precedent for human genetic variation impacting viral entry receptors to confer human resistance, but to our knowledge this has not been reported for bacterial pathogens.

In *Yersinia* species that infect humans, there are several cell-type-specific host receptors that contribute to disease. However, while the characterization of binding of *Y. pseudotuberculosis* invasin to host β-integrin were landmark studies in understanding bacterial entry into host cells,[20,21] invasin is a pseudogene in *Y. pestis.*[22] Similarly, the YadA adhesin is also a pseudogene in *Y. pestis.*[22] Notably, however, the *Y. pestis* type 3 secretion system (T3SS) cap protein (LcrV) binds to FPR1, an immune cell chemotactic receptor, to facilitate delivery of secreted effectors.[9] FPR1 is essential for the delivery of *Y. pestis* effectors and mice lacking FPR1 display resistance to *Y. pestis* infection. Furthermore, interspecies variation in FPR1 contributes to species-specific disease, and within human populations, a naturally occurring nonsynonymous variant in FPR1 (rs5030880) was found to mediate reduced levels of effector translocation.[9] Additionally, CD205 was reported to serve as a dendritic cell entry receptor.[23] Dendritic cells have been shown to traffic *Y.* pestis to the draining lymph node and help spread bacteria through the

This is an open access article under the CC BY license (http://creativecommons.org/licenses/by/4.0/).

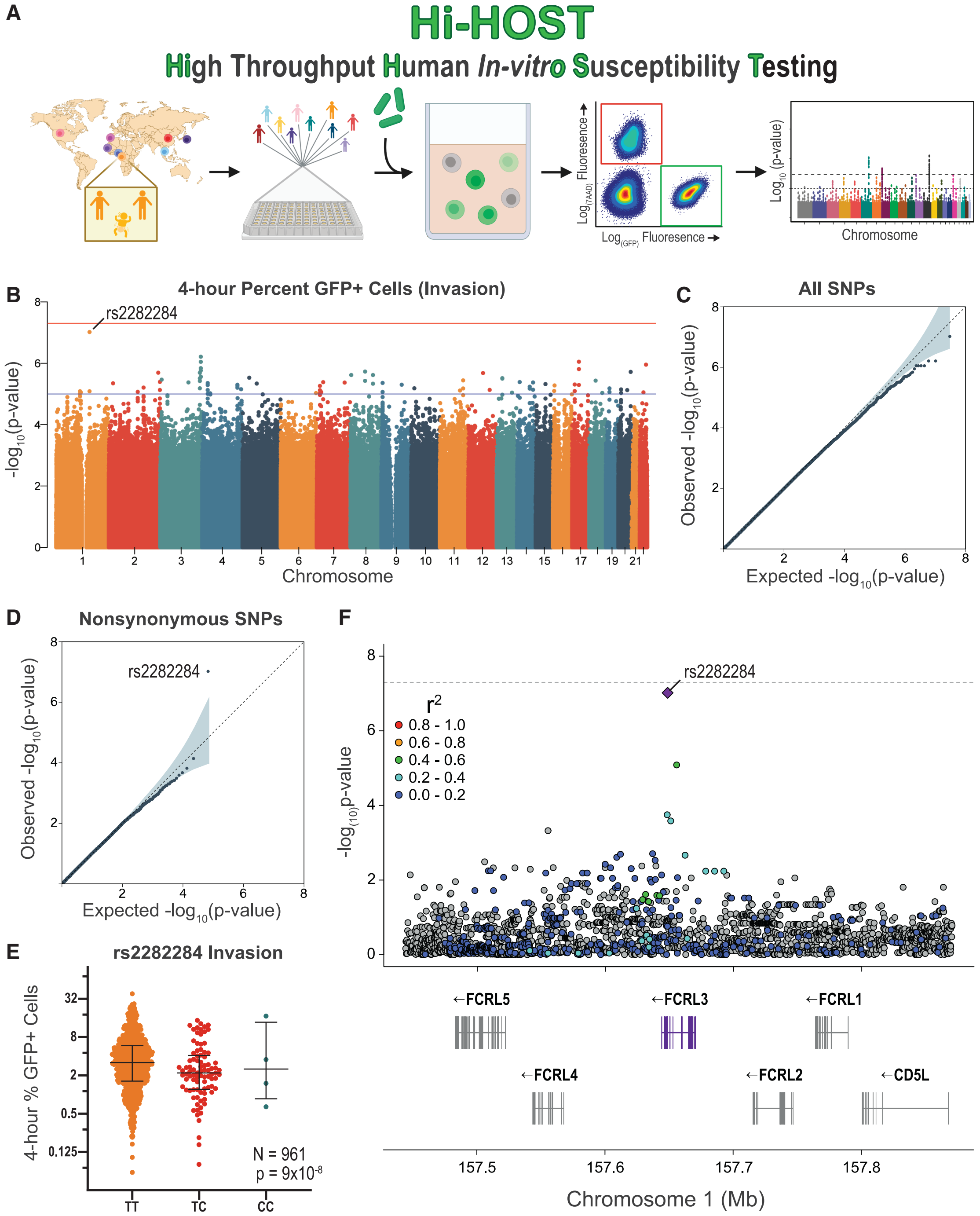

Figure 1. Hi-HOST cellular GWAS of *Y. pestis* invasion

(A) Flowchart of Hi-HOST for *Y. pestis* invasion. The map displays the eight populations tested in this study (CHB, Han Chinese in Beijing, China; KHV, Kinh in Ho Chi Minh City, Vietnam; JPT, Japanese in Tokyo, Japan; GWD, Gambian in Western Division, The Gambia; YRI, Yoruba in Ibadan, Nigeria; ESN, Esan in Nigeria; CEU, Utah residents (CEPH) with Northern and Western European ancestry; IBS, Iberian populations in Spain). The image was partially created with BioRender software.

(B) Manhattan plot of *Y. pestis* invasion measured at 4 hpi (4 h % GFP$^+$ cells) for 961 genetically diverse LCLs after analysis with QFAM-parents using PLINK. Orange and blue lines show suggestive ($p = 1 \times 10^{-5}$) and genome-wide significant ($p = 5 \times 10^{-8}$) thresholds. The lead SNP on chromosome 1 is rs2282284.

(C) Quantile-quantile plot of observed vs. expected $-\log(p$ values) for all 15,213,612 SNPs demonstrates minimal deviation from the null distribution (gray line).

(D) Stratified quantile-quantile plot for 33,723 nonsynonymous SNPs reveals a single SNP with a p value lower than expected by chance (rs2282284; $p = 9 \times 10^{-8}$).

(E) Genotypic mean plot of rs2282284 for *Y. pestis* invasion in 961 LCLs. Bars indicate the mean and interquartile range. The C allele is associated with a lower percentage of infected cells at 4 h ($p = 9 \times 10^{-8}$) by QFAM-parents analysis.

(legend continued on next page)

lymphatic system.[7,24] Most work on *Y. pestis*-host interactions has focused on readily phagocytic cells like neutrophils, macrophages, and dendritic cells without more closely examining a large component of lymphatic organs: lymphoid lineage cells. While *Y. pestis* is found to alter gene expression and interact with these cells during infection,[10,25,26] there are no recognized *Y. pestis* receptors specific to B and T cells.

Here, we used a cellular GWAS approach called Hi-HOST (high-throughput human *in vitro* susceptibility testing)[27–29] to model a "pandemic-in-a-plate" and harness global natural human genetic diversity to discover variation that regulates *Y. pestis* invasion into lymphoblastoid cell lines (LCLs; Epstein-Barr virus-immortalized B cells). We identified a nonsynonymous variant (rs2282284) in *Fc receptor-like 3* (*FCRL3*) associated with host cell entry of *Y. pestis* that does not require opsonization. We show that FCRL3 and other FCRL proteins, including FCRL5, facilitate attachment and invasion of *Y. pestis* and that rs2282284 severely impairs a motif that influences kinase-mediated invasion of human cells. Thus, human genetic variation has revealed a lymphocyte-specific receptor for *Y. pestis.*

RESULTS

A cellular GWAS of *Y. pestis* infection

To uncover human genetic differences that impact interactions with *Y. pestis*, we applied Hi-HOST to *Y. pestis* host cell invasion and early survival (Figure 1A). This system presented an opportunity to identify common human variants that impact general host factors in *Y. pestis* infection, as well as specific factors that may play a critical role in the lymphatic tropism of early disease. Briefly, a modified gentamicin-protection assay using *Y. pestis* KIM6$^+$ tagged with isopropyl β-D-1-thiogalactopyranoside (IPTG)-inducible GFP was coupled with flow cytometric quantification of the percentage of GFP$^+$ cells at 4 h post-infection (hpi; referred to as "invasion"). KIM6$^+$ lacks the pYV/pCD1 virulence plasmid that encodes the T3SS that is known to reduce uptake into host cells,[30–33] creating an environment similar to early mammalian infection before activation of the *Yersinia* virulence plasmid. Invasion of *Y. pestis* was broadly inhibited by the presence of serum (Figure S1A), so the assay was conducted in serum-free media. Hi-HOST was carried out on 961 HapMap[34,35] and 1000 Genomes Project[36,37] LCLs from 8 global populations, and each LCL was measured in 3 independent experiments (Table S1). Inter-individual variation was highly reproducible (repeatability = 0.90, 95% confidence interval [CI] = 0.89–0.91), and a substantial fraction of this variation could be attributed to genetic differences based on two methods of heritability estimation ($h^2 = 0.19$ by parent-offspring regression, $p = 0.014$; $h^2 = 0.19$ by genome-wide SNPs, $p = 9.46 \times 10^{-4}$; Figure S1B).

In addition to invading LCLs, *Y. pestis* invasion was observed to occur in primary B cells. Intracellular *Y. pestis* has previously been reported in monocytes[30,39,40] and dendritic cells,[7,24] while bacterial association has been reported with most immune cell types in mice, with up to 20% of infected cells in the inguinal lymph node being B cells at 24 hpi.[26] In fact, infection of peripheral blood mononuclear cells (PBMCs) demonstrated that *Y. pestis* preferentially invaded B cells (CD19$^+$) compared to T cells (CD3$^+$; Figure S1C) with 5 or 18 times greater invasion in PBMCs from two different donors (Figure S1D). Thus, infection of primary human immune cells with *Y. pestis* demonstrated that B cells are preferred targets for invasion *ex vivo*.

We conducted a family-based GWAS on all variants with minor allele frequency (MAF) >1% (QFAM-parents in PLINK[41,42]), leveraging parent-offspring trios to control for population structure (full summary stats available at the Duke Research Data Repository: https://doi.org/10.7924/r43n2d008). Analysis of the quantile-quantile plot revealed no deviation of $-\log_{10}$(p values) from the null distribution (λ = 1). While there was an absence of genome-wide significant hits ($p < 5 \times 10^{-8}$; Figures 1B and 1C), post hoc analysis stratifying GWAS by variant annotations that are more likely to be functional (e.g., nonsynonymous, 5′ or 3′ UTR, expression quantitative trait loci [eQTL]) has proven to be an effective strategy for finding true positive associations.[43] In fact, stratifying for nonsynonymous variants revealed a single SNP, rs2282284, highly deviated from the expected null distribution for this subset of SNPs (Figure 1D). Here, the minor allele C was associated with lower invasion of *Y. pestis* ($p = 9 \times 10^{-8}$; Figure 1E). This nonsynonymous SNP is within *FCRL3*, a gene encoding a member of the FCRL family[44,45] in a region on chromosome 1 that has undergone paralogous expansion to include 6 structurally similar transmembrane FCRL receptors (Figure 1F). Stratification of the GWAS by continental ancestry showed that the phenotype at rs2282284 was primarily driven by the African-ancestry LCLs (due to the higher frequency of the minor C allele), although the directionality of effect was the same in all populations (Figure S2). Interestingly, *FCRL3* is also moderately induced during *Y. pestis* infection of PBMCs, specifically B cells (1.19-fold at 5 h, $p = 0.0019$).[10]

FCRL3 stimulates *Y. pestis* attachment and invasion

FCRLs are a family of type I transmembrane glycoproteins expressed primarily on B cells.[44,45] While they are structurally similar to the antibody binding Fc receptors (FcRs), only recently has the direct binding capacity of some FCRLs to specific immunoglobulin (Ig) classes been biochemically demonstrated.[46,47] Other FcRs have been reported to serve as entry receptors for cytomegalovirus and Enterovirus B,[48,49] and phagocytosis of opsonized bacteria can also be triggered by FcRs.[50–52] However, invasion of LCLs by *Y. pestis* in Hi-HOST was specifically measured in the absence of serum, meaning the bacteria was unopsonized during interaction with FCRL3. Following ligand binding, FcRs utilize immunoreceptor tyrosine-based signaling motifs to trigger cellular processes, including receptor-mediated endocytosis and phagocytosis.[53–56] FCRL3 has one immunoreceptor tyrosine-based activation motif (ITAM; amino acids 650–665: Y_{650}SNVNPGDSNPIY$_{662}$SQI) that is known to activate

(F) A local Manhattan plot displaying a 200-kb flanking region of *FCRL3* on chromosome 1 demonstrates that rs2282284 is within the gene. A purple diamond denotes rs2282284 and SNP LD is shown by colors as indicated; gray has no LD data. LD was determined using data from all populations in the study based on combined LDLink data accessed through R package locuszoomr.[38]
See also Figures S1 and S2 and Table S1.

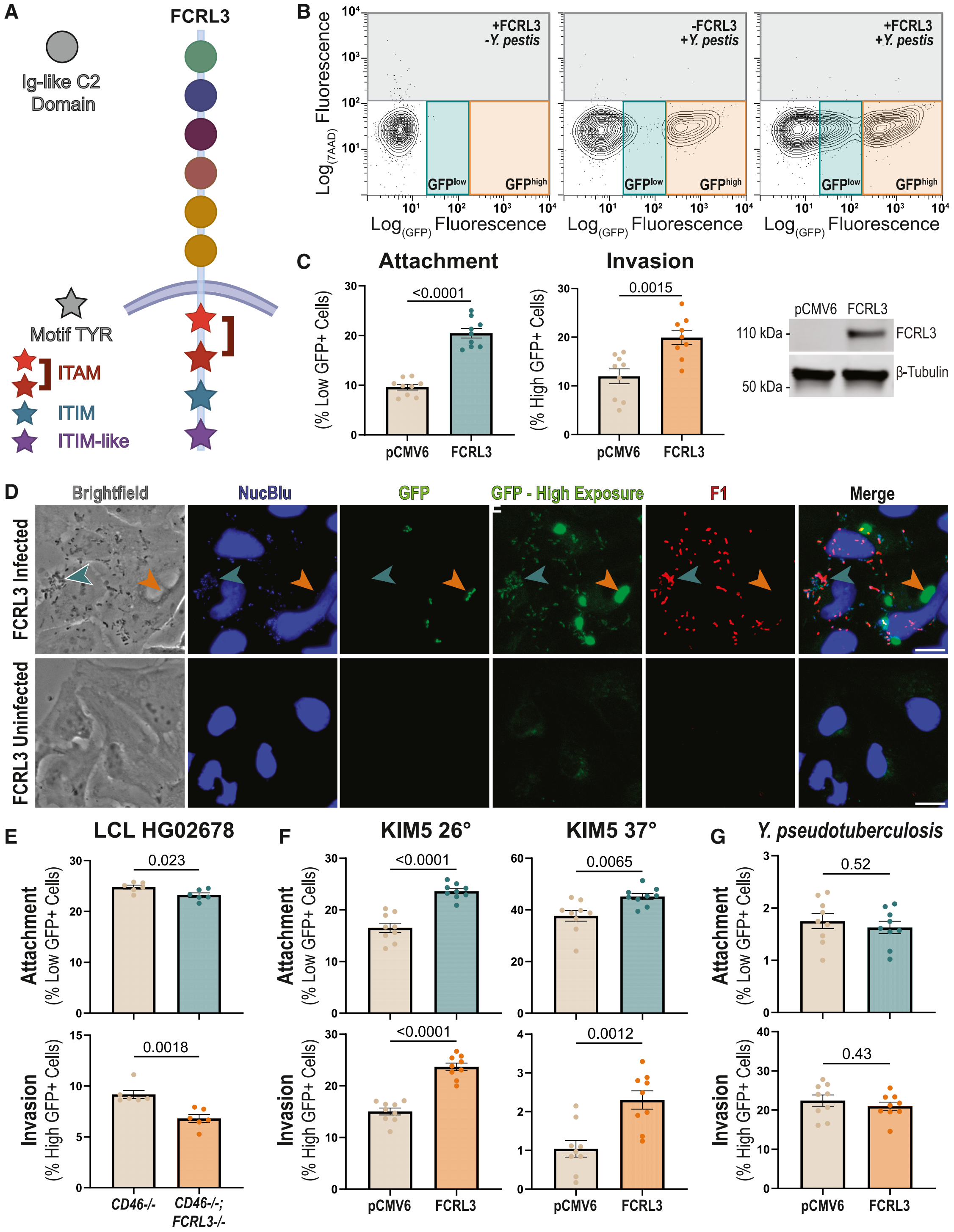

Figure 2. FCRL3 stimulates *Y. pestis* invasion

(A) Diagram of FCRL3 protein. Ig-like C2 domains are depicted as circles, with the color indicating their phylogenetic relationship, while tyrosines involved in intracellular signaling motifs are shown as stars. Red indicates an ITAM, light blue an ITIM, and purple indicates the ITIM-like/HemITAM Y722 motif.

For (B)–(D), (F), and (G), HeLa cells were transfected with empty vector or *FCRL3* plasmid. For (B)–(F), cells were infected using a gentamicin protection assay, and then GFP was induced in living, intracellular bacteria for 2 h with IPTG. (B) Flow cytometry of *Y. pestis* infection of HeLa cells. After infection, two populations of GFP^{+} cells were detected: HeLa cells infected with living, intracellular bacteria (GFP^{high}, orange) and those with attached, dead bacteria (GFP^{low}, light blue). (C) Overexpression of FCRL3 increases attachment (GFP^{low}, light blue) and invasion (GFP^{high}, orange). Three biological replicates in each of three experiments were

(legend continued on next page)

internalization in other FcRs,[57] and one immunoreceptor tyrosine-based inhibitory motif (ITIM; amino acids 690–695: VLYSEL) (Figure 2A).[55] Additionally, the nonsynonymous SNP rs2282284 causes an asparagine-to-serine change at FCRL3 residue 721, directly next to an additional phosphotyrosine (Y722) in an ITIM-like motif (amino acids 719–725: EENYENV), which is thought to associate with Src homology 2 domain-containing phosphatases but does not completely recapitulate ITIM phenotypes or sequence homology.[55,58] In fact, this motif may be acting as an ITAM-like motif, perhaps in the way of a HemITAM (DED/EGYxxL), which are highly plastic in their function based on the amino acids they contain.[59] Therefore, we hypothesized that FCRL3 might be serving as a cell surface receptor for *Y. pestis*, and rs2282284 might interfere with the cytosolic signaling required to mediate uptake of FCRL3 bound to *Y. pestis.*

Previous studies have commonly used heterologous expression in non-phagocytic cells as evidence of the ability of cell surface proteins to function as phagocytic receptors.[60–62] To assess whether FCRL3 is sufficient to mediate the attachment and invasion of *Y. pestis*, we overexpressed *FCRL3* in HeLa cells, which allows for simpler interpretation as they have no detectable endogenous FCRL3 expression (Figure S3A). Careful examination of these cells by flow cytometry revealed two distinct GFP^{+} populations after washing away non-adhered bacteria, defined as GFP^{high} and GFP^{low} (Figure 2B). Overexpression of *FCRL3* substantially increased both GFP^{high} and GFP^{low} populations (Figure 2C). Using fluorescence microscopy, we discovered that the GFP^{high} population are host cells infected with living intracellular bacteria that are protected from gentamicin killing and express high levels of GFP in response to IPTG. These bacteria are found within a $LAMP1^{+}$ *Yersinia*-containing vacuole (YCV) (Figure S3B). We speculated that the GFP^{low} population were host cells with adherent, extracellular bacteria with low but detectable baseline GFP expression that had been killed after the addition of gentamicin. To confirm that these adherent GFP^{low} bacteria were indeed extracellular *Y. pestis*, we stained non-permeabilized cells using an antibody for the F1 capsule (found on the surface of *Y. pestis* after induction at 37°C).[63] F1 antigen was detected exclusively on the surface of bacteria with low GFP expression (Figure 2D). Thus, the GFP^{low} and GFP^{high} gates are measures of *Y. pestis* attachment and invasion, respectively, and overexpression of FCRL3 in HeLa cells caused significant increases in both phenotypes.

To determine whether FCRL3 is also necessary for attachment and invasion, we created a pooled knockout (KO) of *FCRL3* in LCL HG02678 using CRISPR-Cas9.[64,65] Disruption of *FCRL3* caused a moderate but significant decrease in attachment and invasion (Figure 2E), indicating that FCRL3 is mediating a measurable fraction of total attachment and invasion.

Next, we interrogated the specificity of this process. $KIM6^{+}$, the strain used in Hi-HOST, is attenuated through loss of the pCD1 virulence plasmid. To determine whether pCD1 impacted the FCRL3 phenotypes, assays were repeated with KIM5, a strain that harbors pCD1.[66] KIM5 was grown under conditions that either stimulated (37°C) or suppressed (26°C) the expression of the T3SS from pCD1. As expected, induction of the T3SS resulted in much lower levels of attachment and invasion, but FCRL3 overexpression still led to an increase in both phenotypes when KIM5 was grown at either temperature (Figure 2F). Thus, FCRL3-mediated invasion is independent of pCD1.

Y. pestis evolved from *Y. pseudotuberculosis*, an enteric pathogen,[67] but has undergone both substantial gene loss and acquisition, including two *Y. pestis*-specific plasmids, pMT1 and pPCP1.[68–70] To determine whether FCRL3-mediated invasion was specific for *Y. pestis*, we infected HeLa cells overexpressing FCRL3 and measured the differences in attachment and invasion by flow cytometry. FCRL3 overexpression had no effect on *Y. pseudotuberculosis* invasion (Figure 2G).

Furthermore, we hypothesized that FCRL3-mediated attachment and invasion may be inhibited by the presence of serum. This was based on the reported binding of FCRLs to antibodies[46,47,71] and our observation that most *Y. pestis* invasion into LCLs was inhibited by fetal bovine serum (FBS) (see Figure S1). Surprisingly, while most invasion into HeLa cells was blocked by 10% FBS, the FCRL3-dependent increase remained (Figure S3C). This demonstrates that while much of *Y. pestis* invasion is blocked by components of serum, FCRL3-dependent invasion occurs in a serum-independent manner.

FCRL3 colocalizes with *Y. pestis* at sites of bacterial attachment

Following ligand binding, FcRs cluster together before the activation of intracellular signaling.[72] We hypothesized that *Y. pestis* binding to FCRL3 might trigger a similar process and tested this

plotted, and an unpaired t test was performed to determine significance. A western blot was performed to confirm the presence of FCRL3 after overexpression using 1:200 FcRH3 antibody (Santa Cruz, C-2). (D) GFP^{low} cells have attached, extracellular *Y. pestis.* Infected HeLa cells were fixed with 4% paraformaldehyde (PFA) after 4 h. Infected and uninfected cells were blocked with normal goat serum (but not permeabilized) and then incubated with 1:20 polyclonal anti-*Y. pestis* F1-antigen antibody (BEI Resources, NR-31024) to stain extracellular bacteria. DNA was stained with NucBlue for nuclei visualization. To visualize the GFP^{low} bacteria, a high- and low-exposure GFP panel is shown. GFP^{low} bacteria are indicated with a teal arrowhead and GFP^{high} bacteria with an orange arrowhead. Cells were imaged using a 40× air objective on an EVOS M5000 microscope. ImageJ was used to adjust and crop images and to add scale bars (20 μm).
(E) CRISPR-mediated knockout (KO) of *FCRL3* in LCL HG02678 decreases attachment and invasion of *Y. pestis*. Cells were electroporated with either only *CD46* (control) or *FCRL3* + *CD46* guides and CRISPR-Cas9. Pooled KO cells (>65% $FCRL3^{-/-}$) were sorted for $CD46^{-/-}$ cells. Three experiments with two biological replicates of each condition were plotted, and an unpaired t test was performed to determine significance. (F) *FCRL3* overexpression increases attachment and invasion of *Y. pestis* KIM5. KIM5 was subcultured for 2 h 40 min at either 26°C or 37°C before infecting HeLa cells. Three biological replicates in each of three experiments were plotted, and an unpaired t test was performed to determine significance.
(G) FCRL3 overexpression has no effect on *Y. pseudotuberculosis* attachment and invasion. Infected HeLa cells were assayed for attachment and invasion by flow cytometry at 4 hpi. Three biological replicates in each of three experiments were plotted, and an unpaired t test was performed to determine significance.
(C and E–G) Mean ± SEM is shown. Experiments were normalized by grand mean.
See also Figure S3.

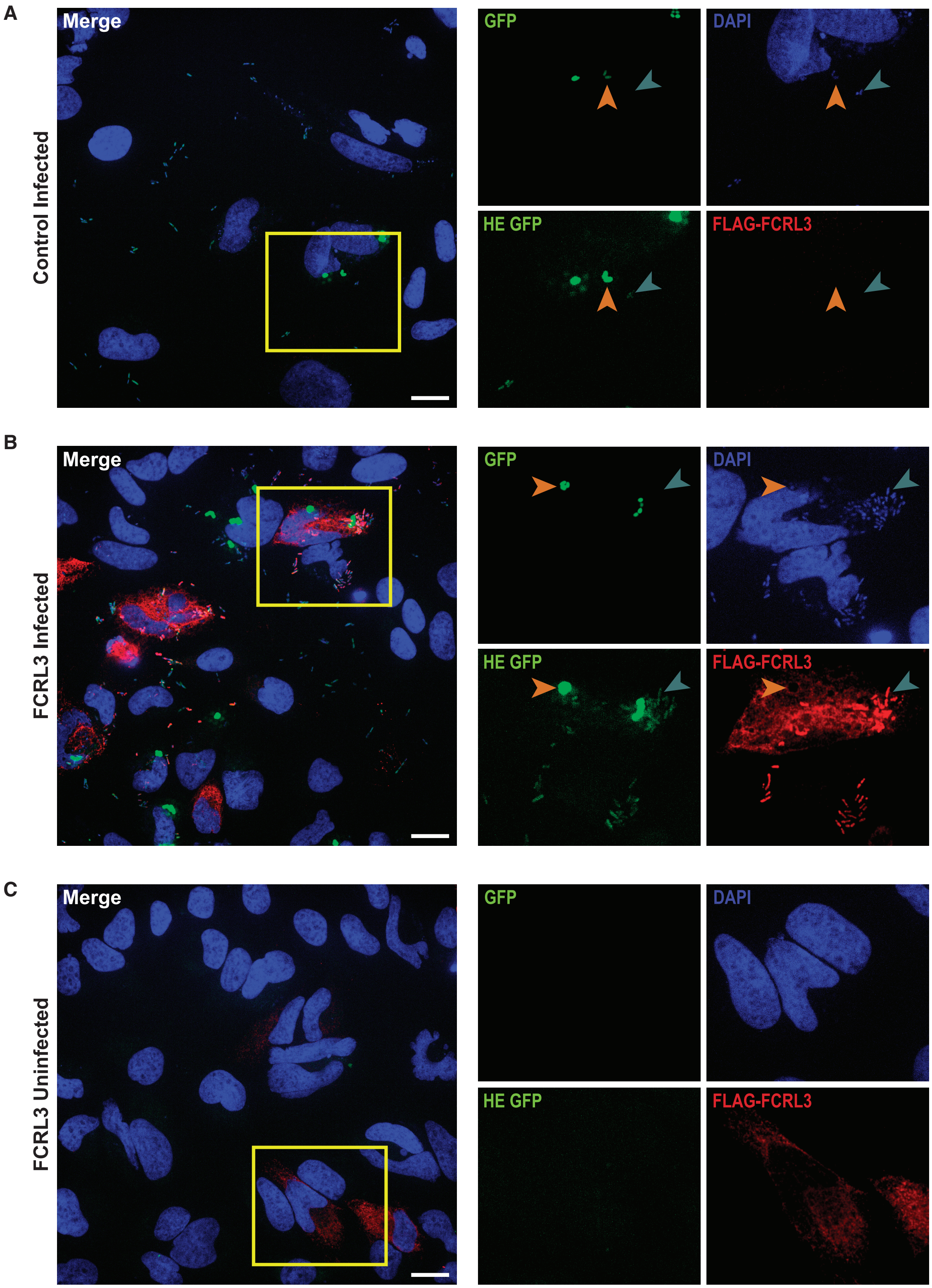

Figure 3. FCRL3 colocalizes with *Y. pestis* at sites of bacterial attachment

(A–C) HeLa cells transfected with empty vector (A) or *FCRL3* plasmid (B) were infected with KIM6^{+} +p67GFP3.1 *Y. pestis* by gentamicin protection assay and GFP was induced with IPTG for 2 h prior to fixation with 4% PFA. *FCRL3* transfected uninfected control is shown in (C). For (A)–(C), after incubating for 30 min in block/perm, DNA was stained with 2.5 μM DAPI, FLAG-FCRL3 was stained red using DYKDDDDK Tag antibody (Cell Signaling Technology, D6W5B), and GFP^{+} bacteria

(legend continued on next page)

through localization studies of overexpressed FLAG-tagged FCRL3 in HeLa cells. FLAG-FCRL3 protein primarily exhibited diffuse localization in uninfected cells. Remarkably, following exposure to *Y. pestis*, FLAG-FCRL3 colocalized with GFP^{low} (but not GFP^{high}) *Y. pestis* (Figure 3). This indicates that FCRL3 undergoes clustering to sites of *Y. pestis* attachment but that it is generally not present in the YCV. Thus, we have demonstrated FCRL3-mediated attachment and invasion in HeLa cells with clustering of FCRL3 at sites of attachment. Defining these phenotypes facilitated testing structurally and functionally similar members of the FCRL family and subsequent mutagenesis studies.

Functional redundancy for *Y. pestis* attachment and invasion among FCRL proteins

While FCRL3 overexpression in HeLa cells was sufficient to induce attachment and invasion, attachment or invasion was only moderately decreased in *FCRL3*$^{-/-}$ LCLs (see Figure 2E). We hypothesized that the presence of the five other transmembrane FCRL family proteins in humans and hundreds of proteins in the Ig superfamily might lead to redundancy.

Each FCRL protein is structurally similar, containing a mix of five phylogenetically related types of extracellular Ig-like C2 domains and intracellular motifs that mediate cell signaling (Figure 4A).[44,73] These proteins have considerable plasticity in the number, type, and location of the domains and motifs. We therefore hypothesized that this natural genetic variation might cause phenotypic variation in *Y. pestis* attachment and invasion. After overexpressing each available transmembrane construct (FCRL1, FCRL3–6)[47] in HeLa cells and infecting, only FCRL1 was unable to significantly increase attachment compared to an unrelated Ig-like domain containing receptor CD31 (PECAM-1) (Figure 4B). The first and/or second Ig-like C2 domains of FCRL3 were present in all FCRL proteins that increased *Y. pestis* attachment (see Figure 4A). Of those FCRLs that promoted attachment, only FCRL3 and FCRL5 significantly increased invasion (Figure 4B). This property coincides with the presence of an ITAM (see Figure 4A), which is reported to interact with the kinases SYK and Zap70 to activate cellular processes including endocytosis and phagocytosis.[55] Interestingly, we found that while SYK overexpression alone had no effect on invasion into HeLa cells, co-expression with FCRL3 resulted in a significant increase compared to FCRL3 alone (Figure 4C). Additionally, an SYK inhibitor (BAY 61-3606) inhibited *Y. pestis* invasion into LCLs (HG02678 and GM19204) (Figure 4D). Thus, there are shared features among FCRL proteins that may mediate attachment (Ig-like domains 1 and 2) and invasion (an ITAM motif that signals through SYK), ultimately resulting in functional redundancy within the FCRL family.

Mutational analysis of FCRL-mediated attachment and invasion of *Y. pestis*

The FCRL proteins that can mediate bacterial attachment (FCRL3–6) have Ig-like C2 domains with similarity to Ig-like domain (IgLD)-1 and/or IgLD-2 of FCRL3. Therefore, to determine whether these Ig-like domains were required for binding, we created plasmids with deletions of each as well as IgLD-3 for comparison. We observed a significant decrease in attachment relative to WT in all three mutants, but the effect was largest with IgLD-1 deletion. Additionally, only IgLD-1 deletion significantly decreased invasion (Figure 5A). To further investigate this phenomenon, we inserted IgLD-1 into FCRL1, which does not cause a significant increase in attachment or invasion when overexpressed (see Figure 4B). Insertion of IgLD-1 into FCRL1 was sufficient to significantly increase attachment and invasion, although not to the level of FCRL3 (Figure 5B). The partial increase could be due to the lower protein abundance of the mutant.

Downstream effects of FcRs are canonically mediated by phosphorylated ITAMs or ITIMs and their interaction with kinases such as SYK.[56] We found that the FCRLs with ITAM motifs were able to facilitate *Y. pestis* invasion (see Figure 4B). Additionally, FCRL3 has a motif at Y722 that imperfectly matches the ITIM consensus.[58] The missense mutation caused by rs2282284 (N721S) is directly adjacent to the tyrosine in this ITIM-like/possible HemITAM. We created mutants containing tyrosine-to-phenylalanine mutations and a mutant with the rs2282284 N721S mutation. In HeLa cells, co-overexpression of the mutant constructs and SYK followed by infection revealed that the mutated ITAM (Y650F; Y662F) significantly decreased FCRL3-dependent invasion, the mutated ITIM (Y692F) had no effect, and Y722F (within the ITIM-like/HemITAM) caused the same reduction in SYK-mediated invasion as the mutated ITAM construct (Figure 5C). In fact, altering the rs2282284 T allele to the minor C allele (N721S; associated with lower invasion) caused a reduction in invasion similar to that of Y722F. This indicates the Y722 motif promotes SYK and FCRL3-dependent *Y. pestis* invasion, and the N721 position is critical for function. This finding further suggests the Y722 motif may be acting as a HemITAM in FCRL3.

To test the importance of these mutations on FCRL3 clustering and SYK recruitment, we first monitored colocalization of FCRL3, SYK, and *Y. pestis* in HeLa cells overexpressing FCRL3 at 15, 30, and 60 min post-infection. SYK and FCRL3 colocalization with attached *Y. pestis* increased throughout this time course when co-expressed (Figure 5D). Therefore, FCRL3 clusters at sites of *Y. pestis* attachment, and SYK is recruited to these sites. We then assessed the effects of the intracellular signaling (ITAM, Y722F, and N721S) and extracellular attachment (IgLD-1) mutations at the 1-h time point. As predicted, the IgLD1 deletion led to a complete ablation of FCRL3 clustering, and a reduction in SYK recruitment. Interestingly, N721S resulted in a greater but not statistically significant fraction of SYK colocalized with FCRL3 and *Y. pestis* (Figure 5E). This was unexpected given the reduced attachment and invasion of this mutant. We speculate, however, that SYK can bind to N721S but is unable to efficiently trigger phagocytosis or other downstream steps.

are shown at low and high exposure to demonstrate the GFP^{low} bacteria. GFP^{low} bacteria are indicated with a teal arrowhead and GFP^{high} bacteria with an orange arrowhead. The individual fluorescence channels for the portion of the merged image outlined by the yellow boxes are shown enlarged on the right. Images were taken using a Crest X-light V2 spinning disk confocal system (CrestOptics) on a Zeiss Observer Z1 inverted microscope with a 63× water objective. ImageJ was used to adjust and crop images and to add scale bars (20 μm).

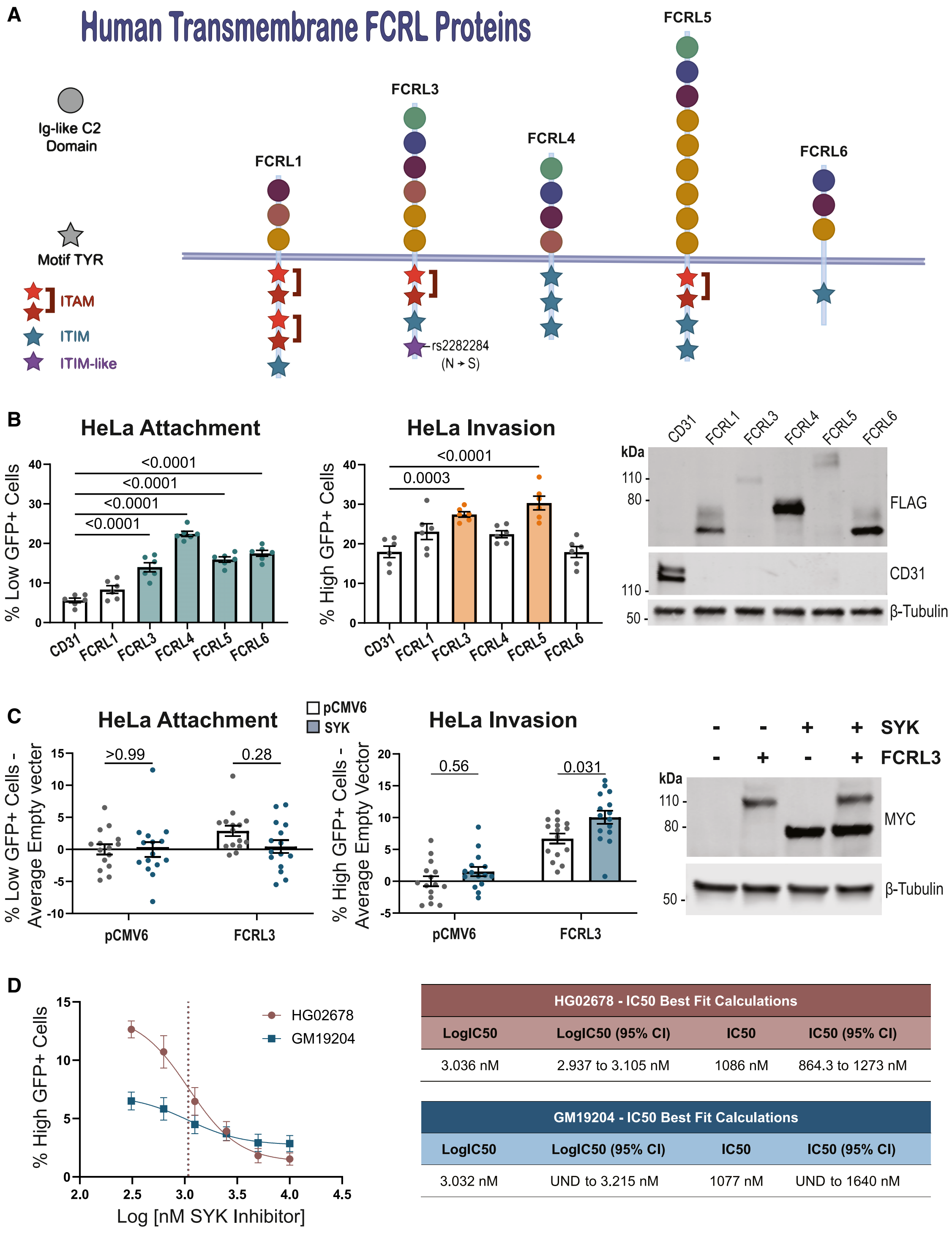

HG02678 - IC50 Best Fit Calculations			
LogIC50	LogIC50 (95% CI)	IC50	IC50 (95% CI)
3.036 nM	2.937 to 3.105 nM	1086 nM	864.3 to 1273 nM

GM19204 - IC50 Best Fit Calculations			
LogIC50	LogIC50 (95% CI)	IC50	IC50 (95% CI)
3.032 nM	UND to 3.215 nM	1077 nM	UND to 1640 nM

Figure 4. Determinants of attachment and invasion across FCRL paralogs

(A) Protein homology of the human transmembrane FCRL proteins used in this study. Ig-like C2 domains are indicated by a circle and are colored by their phylogenetic relationship with one another. Tyrosine motifs are indicated by stars with red, blue, or purple corresponding to ITAM, ITIM, or ITIM-like/HemITAM Y722 motif, respectively.

(B) Overexpression of *FCRL*s have varying effects on attachment and invasion. HeLa cells were transfected with the indicated plasmid and assayed for attachment and invasion by flow cytometric gentamicin protection assay at 4 hpi. Colored boxes indicate FCRLs that are capable of increasing attachment (light blue) or invasion (orange). Two experiments with three biological replicates of each condition were plotted, and a one-way ANOVA with Dunnett's multiple

(legend continued on next page)

Direct binding of FCRL proteins to *Y. pestis*

The FCRL family impacts attachment and invasion of *Y. pestis* into cells, and this could be due to direct binding or could be secondary to effects of FCRLs on other cell surface molecules. To test this, we measured direct binding using the purified extracellular domain of the structurally and functionally similar FCRL5 fused to COMP5AP-AviTag-9xHis.[74] The FCRL5 fusion protein was expressed at substantially higher levels than its FCRL3 counterpart, and overexpression of FCRL5 in HeLa cells was sufficient for stimulating attachment and invasion (see Figures 4B and 4C), so it was used for direct binding experiments. The 9xHis tag facilitated purification, while the human placental alkaline phosphatase (AP) allowed for enzymatic detection of protein. A single-step affinity purification resulted in high purity and 13-fold increase in AP specific activity (Figures 6A and 6B). Following purification, FCRL5 fusion or negative control protein (CD31-COMP5AP-AviTag-9xHis) was incubated with *Y. pestis* for 30 min at 4°C, washed with high salt (600 mM NaCl), and *Y. pestis* and bound proteins were lysed and resolved by SDS-PAGE. Due to high levels of endogenous AP from *Y. pestis,* binding was measured by western blot. We observed that 38.8% of FCRL5 fusion protein bound to *Y. pestis* (4.6 times more than to CD31; p = 0.014) (Figures 6C and 6D). Thus, *Y. pestis* directly binds to FCRL proteins.

Impact of rs2282284 on other diseases: Chronic hepatitis C

While *Y. pestis* does not currently pose the threat to civilization that it did in the past, we hypothesized that rs2282284 might have additional consequences for current human health due to its role as an immune receptor. While there are no known genome-wide significant associations for rs2282284, phenome-wide association study (PheWAS) analysis of rs2282284 using BioBank Japan Pheweb[75,76] showed a single trait with a p value less than expected by chance out of over 258 phenotypes: chronic hepatitis C virus infection (Figures 7A and 7B; $p = 9.6 \times 10^{-5}$; $\beta = -0.18$; n = 7,110 cases, 169,588 controls).[76] The C allele (associated with reduced *Y. pestis* invasion) is associated with a reduced risk of chronic hepatitis C. Colocalization analysis with the R package coloc[77] comparing the signals for *Y. pestis* invasion and chronic hepatitis C provided support for the two being due to the same causal variant (Figures 7C–7E; PP4 = 0.78).

DISCUSSION

Using a cellular GWAS of nearly 1,000 LCLs from diverse populations and functional characterization, we have determined that FCRL proteins are direct binding receptors for *Y. pestis*. While some FCRL proteins have recently been demonstrated to bind to Igs,[46,47] a direct role of these proteins in pathogen uptake has not been described. We report that FCRL-mediated uptake of *Y. pestis* consists of (1) binding of *Y. pestis* to the extracellular domain with a requirement for Ig-like domain 1, (2) clustering of FCRL at sites of attachment, and (3) SYK-dependent internalization mediated by ITAMs and ITIM-like/HemITAMs. As these steps mirror how FcRs induce phagocytosis of opsonized bacteria,[60] the question arises as to why *Y. pestis*, a highly adapted, human-specific pathogen, would have developed this means of invasion after evolving from *Y. pseudotuberculosis*,[67] which we found does not utilize FCRL-mediated invasion. We speculate that during this evolution, *Y. pestis* has evolved mechanisms to hide from the immune system during lymphatic spread. While *Y. pestis* is phagocytosed by professional phagocytic cells, including by FcRs after bacterial opsonization,[78] neutrophils and macrophages are intrinsically more bactericidal than lymphocytes. Therefore, direct FCRL-mediated phagocytosis of *Y. pestis* may provide an intracellular niche within B cells, some of the most abundant cells in the lymphatic system, while also present in low numbers in the skin.[79] Temporally, we hypothesize that this FCRL interaction would be most important soon after injection, prior to expression of the T3SS, which is induced at 37°C,[80] and when the bacteria is susceptible to neutrophil-mediated killing. Once this T3SS is induced, it recognizes the FPR1 host receptor,[9] targeting neutrophils for effector translocation and killing so the bacteria can proliferate.[9] In contrast, *Y. pestis* that takes advantage of an intracellular B cell niche would be shielded from early interactions with neutrophils and other immune cell types until the T3SS can be turned on. Future studies will determine whether this niche might facilitate dissemination from skin to lymph nodes or other FCRL-mediated signaling events that alter infection with *Y. pestis*.

In addition to FCRL3, other FCRL proteins stimulate attachment and invasion. Specifically, the presence of FCRL5, a paralog to FCRL3 with similar attachment and invasion phenotypes, may buffer the effect of the rs2282284 C allele in *FCRL3* on overall *Y. pestis* invasion, despite the mutation severely decreasing SYK-dependent FCRL3 function. This buffering effect of paralogs

comparisons test was performed to determine significance. A western blot was performed to show the presence of each construct using 1:1,000 anti-FLAG (Sigma-Aldrich, M2) or 1:1,000 anti-CD31 (Cell Signaling Technology, 89c2).

(C) Co-expression of *SYK* increases FCRL3-dependent invasion. HeLa cells were transfected with the indicated plasmids and assayed for attachment and invasion by flow cytometric gentamicin protection assay at 4 hpi. *SYK* co-expression is indicated with a blue shaded bar. Five experiments with three biological replicates of each condition were plotted, and a two-way ANOVA was performed with Tukey's multiple comparisons test to determine significance. Grand mean normalized values were used, and the experimental average empty vector (pCMV6 only) value was subtracted from each value from each corresponding experimental replicate. Western blot displays the presence of MYC-SYK and MYC-FCRL3 by 1:200 anti-MYC-Tag (Cell Signaling Technology, 71D10).

(D) SYK inhibition significantly reduces invasion into LCLs. SYK inhibitor was added at 0.31, 0.63, 1.25, 2.5, 5, and 10 μM to LCL HG02678 and GM19204 at 60 min prior to infection with KIM6$^+$. Cells were assayed for attachment and invasion by flow cytometric gentamicin protection assay at 4 hpi. The half-maximal inhibitory concentration (IC50) was determined for each line by nonlinear fit after log transforming the data. For GM19204, the lower confidence interval is undefined. To determine the significance of the observed decrease, an unpaired t test was performed between the lowest (0.31 μM) and highest dose (10 μM) for each LCL. For both HG02678 and GM19204, p values were <0.0001.

Data are from three experiments of three biological replicates for each cell line and condition. Error bars represent mean ± SEM.

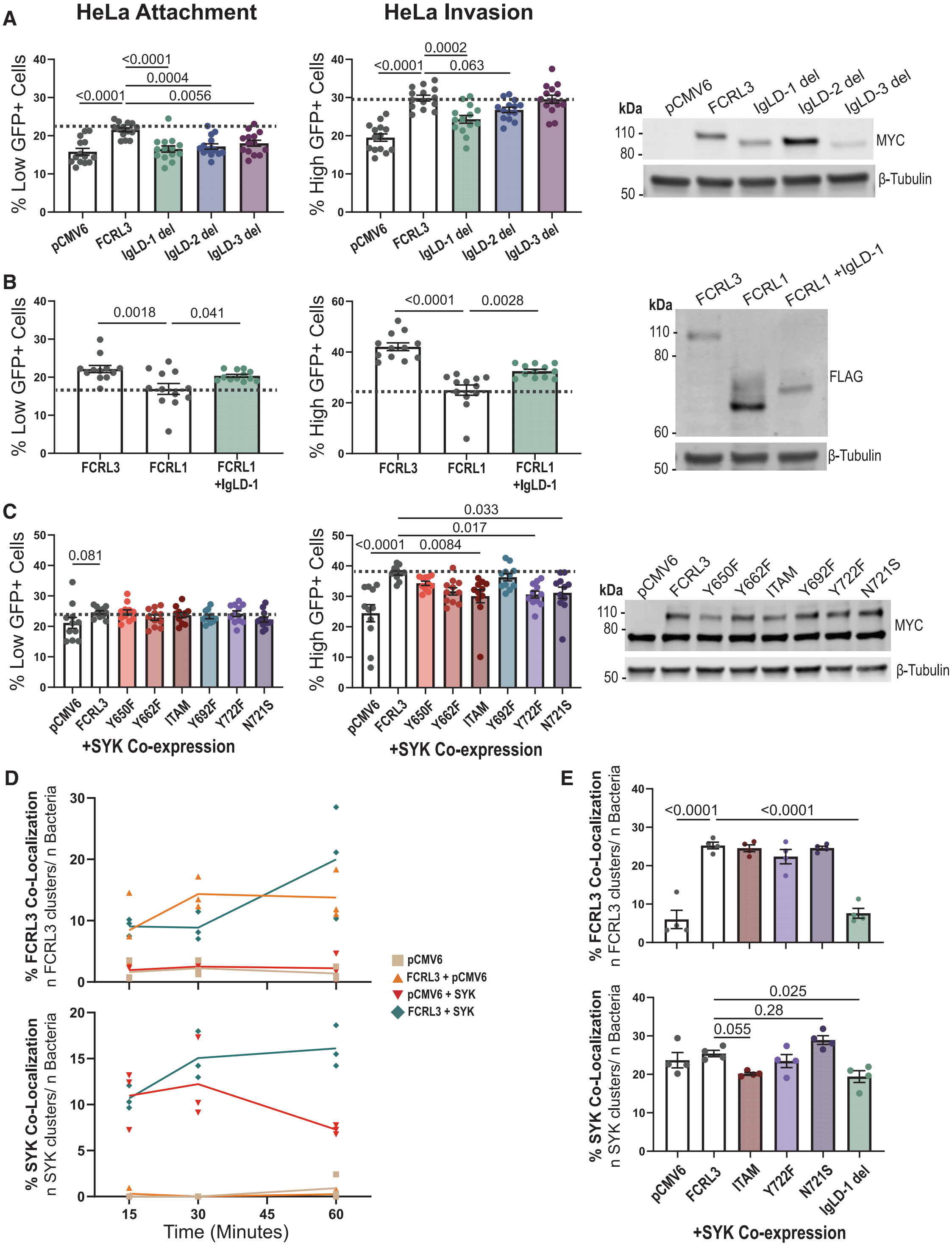

Figure 5. Mutational analysis of FCRL3 reveals the importance of Ig-like domain 1 (IgLD-1) and the ITIM-like/HemITAM Y722 motif containing rs2282284

(A) Deletion of IgLD-1 leads to significant decreases in attachment and invasion. Four experiments with three or five replicates were plotted, and a western blot demonstrates that each of the MYC-tagged constructs are overexpressed with 1:200 anti-MYC-Tag (Cell Signaling Technology, 71D10).

(B) Insertion of IgLD-1 into FCRL1 leads to an increase in attachment and invasion. Four experiments with three replicates were plotted, and western blot demonstrates each of the FLAG-tagged constructs are overexpressed using 1:1,000 anti-FLAG (Sigma-Aldrich, M2).

(legend continued on next page)

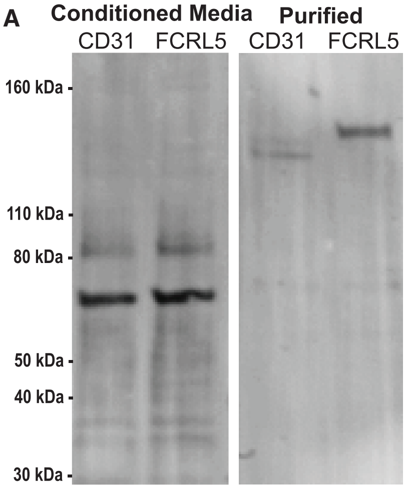

B

Detecting Fold Purification of FCRL5 by Phosphatase Activity

	Total protein (mg)	AP Activity (U)	Specific Activity (U/mg)	Fold Purification
Conditioned Media	0.554	0.312	0.56	-
Purified Elution	0.0045	0.033	7.33	13

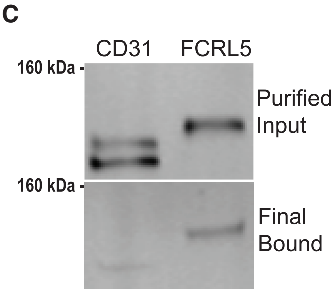

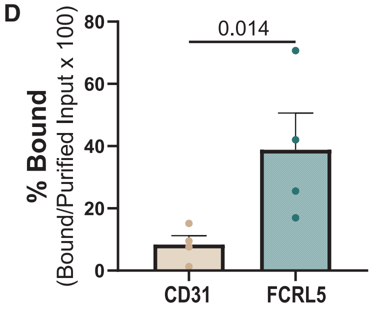

Figure 6. The FCRL5 extracellular domain directly binds to *Y. pestis*

(A) Total protein stain of supernatant from Expi293 cells transfected with CD31-COMP5AP-AviTag-9xHis or FCRL5-COMP5AP-AviTag-9xHis and purified protein following cobalt bead purification.

(B) Table of purification demonstrates increased specific activity after purification. Total protein concentration was measured by Bio-Rad Protein Assay, and AP activity was measured using BluePhos Microwell Substrate Kit (KPL).

(C) Western blot of binding displays purified protein input and protein bound after incubation with *Y. pestis*, two washes, and subsequent lysis and sonication. Blots were stained with 1:1,000 Avi Tag monoclonal antibody (Thermo Fisher).

(D) Bar graph showing quantification of bound vs. purified input western blots using infrared secondary antibodies (Li-COR Odyssey) from four binding experiments. Significance was tested by a paired ratio t test. Mean ± SEM is shown.

likely underlies the observation that human genes that have a paralog are less likely to have severe consequences in human disease.[81] The redundancy likely extends beyond the FCRL family, as the Ig-like C2 domain subtype required for FCRL3-mediated uptake (similar to FCRL3's Ig-like domain 1 or D1 from the nomenclature[44]) is found not only in FCRL5 but also in conventional FCR family members.[44] It remains to be determined whether the ability to facilitate *Y. pestis* binding and entry extends to other FcRs and indeed to the more distantly related members of the Ig superfamily. Previous reports describe two other FcRs that can bind bacteria in the absence of opsonization. CD89 appears to act as an innate immune receptor that binds and triggers phagocytosis and killing of bacteria in macrophages,[82] while CD16A binds to *Escherichia coli* to activate an ITAM-inhibitory pathway that prevents phagocytosis and contributes to sepsis.[83]

The SNP rs2282284 is present in all populations of the 1000 Genomes Project,[37] with a global MAF of 6% (from 2% [Han Chinese in Beijing, China]) to 11% (Indian Telegu in the United Kingdom). Thus, this allele (the derived C allele associated with reduced *Y. pestis* invasion that encodes for the N721S mutation) was present prior to the out-of-Africa expansion, but there does not appear to have been obvious population differentiation at this locus or other signals of positive selection (e.g., singleton density score [SDS][84]; best SDS = 1.40, p = 0.16 in African ancestry individuals from the Trans-Omics for Precision Medicine Program [TOPMed] dataset[85]). Being near 5% MAF may have resulted in exclusion of the SNP in some GWASs, and even when included, low numbers of homozygous C individuals may limit the power to detect significant associations. Despite this, we found that rs2282884 was associated with another infectious disease, chronic hepatitis C virus infection, in Japanese individuals. The same C allele that confers resistance to *Y. pestis* cellular infection also confers protection against chronic hepatitis C virus infection. While hepatitis C virus primarily infects hepatocytes, it has been demonstrated to infect B cells,[86] leading to the suggestion that hepatitis C virus may use B cells as a protective

(C) Intracellular motif mutants lead to decrease in invasion when co-expressed with *SYK*. SYK co-expression is indicated in blue. Four experiments with two or three replicates for each condition are plotted. Western blot displays the presence of MYC-SYK (~75 kDa) and MYC-FCRL3 (~100 kDa) by 1:200 anti-MYC-Tag (Cell Signaling Technology, 71D10).

(D) Time course of FCRL3 and SYK recruitment to sites of GFP^{low} bacteria at 15, 30, and 60 min. HeLa cells transfected with the indicated plasmids were infected with $KIM6^+$ +p67GFP3.1 *Y. pestis* (pre-induced during liquid culture with IPTG 2 h prior to infection) and fixed with 4% PFA at the indicated time. Quantification of three experiments were plotted, and ~50 infected cells were counted for each condition.

(E) Quantification of FCRL3 and SYK clusters at sites of GFP^{low} bacterial attachment for ITAM, Y722F, N721S, and IgLD-1 mutants. After infecting as described in (D), cells were fixed at 1 hpi. Four experiments each quantifying ~50 SYK^+ cells from each condition were plotted.

(A–C) HeLa cells were transfected with the indicated plasmids and assayed for attachment and invasion by flow cytometric gentamicin protection assay at 4 hpi. (D and E) Slides were stained with 1:400 anti-DYKDDDDK Tag (D6W5B) and 1:100 anti-SYK (4D10) (Cell Signaling Technology). The experiment was performed blinded, with one researcher transfecting the cells and another counting the cells after staining. (A–C and E) A one-way ANOVA was performed with Dunnett's multiple comparisons test to determine significance. (A–E) Mean ± SEM is shown. Experiments were normalized using grand mean.

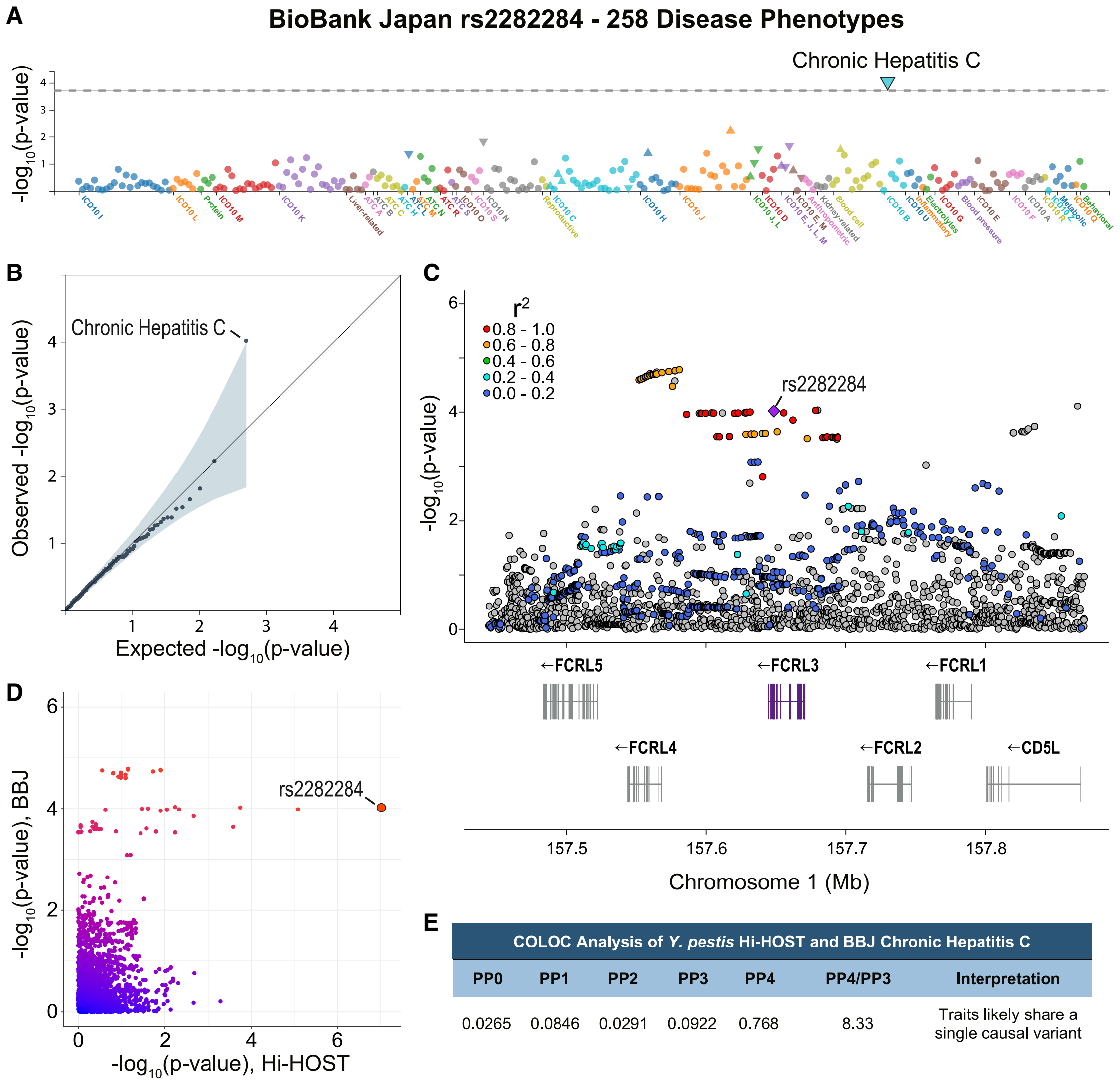

COLOC Analysis of *Y. pestis* Hi-HOST and BBJ Chronic Hepatitis C						
PP0	PP1	PP2	PP3	PP4	PP4/PP3	Interpretation
0.0265	0.0846	0.0291	0.0922	0.768	8.33	Traits likely share a single causal variant

Figure 7. rs2282284 is associated with chronic hepatitis C

(A) PheWAS plot displaying the $-\log_{10}(p)$ values of 258 phenotypes in BioBank Japan PheWeb. Phenotypes are colored by disease group.

(B) All 258 phenotype *p* values for rs2282284 were plotted on a quantile-quantile plot, and chronic hepatitis C infection was the only phenotype that significantly deviated from the neutral distribution (gray line).

(C) Local Manhattan plot of the rs2282284 locus for the chronic hepatitis C virus phenotype. A purple diamond denotes rs2282284, and LD with SNPs in the locus is shown by colors as indicated; gray has no LD data. LD is based on JPT population.

(D) Plot of the $-\log_{10}(p)$ values from BioBank Japan (*y* axis) and Hi-HOST (*x* axis) GWAS.

(E) Coloc analysis suggests that both phenotypes have the same causal SNP. Table shows the posterior probabilities for each of five hypotheses, with PP4 representing the posterior probability that both traits share the same causal variant. PP4/PP3 is >5, making a stronger case for colocalization.

niche during chronic infection.[87] Thus, rs2282284 is pleiotropic, and the derived C allele appears to protect against both *Y. pestis* and chronic hepatitis C, potentially in similar ways.

Due to the low global frequency despite seemingly positive consequences for historic humans, we speculate there might be balancing selective pressures on rs2282284 that have prevented this allele from reaching higher frequencies. These counterbalancing selective pressures might involve inhibitory activity of FCRL3, whereby it interacts with the B cell receptor to inhibit activation.[58] With such a wide array of functionality, it is perhaps surprising that rs2282284 is not associated with more diseases. However, a different SNP, not in LD (rs3761959; LDlink all population r^2 = 0.0474), is an eQTL (GTEX[88]) and protein-QTL[89,90] for FCRL3. For rs3761959, the derived C allele (global allele frequency 54%) is associated with reduced FCRL3 expression and reduced risk of Graves' disease ($p = 2.27 \times 10^{-12}$)[91] and rheumatoid arthritis ($p = 1 \times 10^{-10}$, Europeans)[92] ($p = 1 \times 10^{-8}$, Europeans)[93] ($p = 5.2 \times 10^{-6}$, Japanese),[94] while increasing the risk of multiple sclerosis ($p = 1.0 \times 10^{-8}$, Europeans).[95,96] While this SNP is associated with autoimmunity, we observe no association of rs3761959

CellPress
OPEN ACCESS

with *Y. pestis* invasion in our Hi-HOST dataset (p = 0.89) and modest association with chronic hepatitis C in the Biobank Japan dataset (p = 0.044). These associations of rs3761959 and rs2282284 with different human traits may point to the role of the Y722 motif in a specialized subset of FCRL3 function. The rs2282284 SNP may specifically impair one aspect of phagocytosis or signaling important in these infectious diseases, while the overall expression level may be more important for functions involved in the development of autoimmunity.

In conclusion, we have used a cellular GWAS of *Y. pestis* to reveal the FCRL proteins as previously unrecognized receptors for the causative agent of plague, which have pleiotropic effects on human infectious and autoimmune disease. We anticipate that future studies using Hi-HOST and *Y. pestis* and other ancient and emerging threats will reveal additional mechanisms in host-pathogen interactions and lasting consequences of pandemic pathogens on the human genome.[2]

Limitations of the study

There are technical limitations of the experimental systems used in this study. Hi-HOST screens use lymphoblastoid cell lines, so they can only identify human genetic differences that affect function in a general or B cell-specific manner. Genetic variants that are important for *Y. pestis'* interactions with other cell types or that require interactions of multiple cell types would not be identified by this method. The expression of FCRL proteins in HeLa cells is valuable for quantitatively assessing effects on attachment and invasion, but they are not the native cell type that expresses FCRL proteins. While loss-of-function experiments in LCLs and our genetic association data support the importance of FCRL proteins for *Y. pestis* invasion of B cells, future studies using primary immune cells, organoid models, and mice will be valuable to further investigate the importance of this invasion mechanism. Such studies will also allow for deeper interrogation into the importance of FCRL-mediated invasion during plague pathogenesis.

RESOURCE AVAILABILITY

Lead contact

Further information, as well as plasmids and bacterial strains generated for this study, are available by request from the lead contact, Dennis C. Ko (dennis.ko@duke.edu).

Materials availability

Plasmids and bacterial strains, as listed in the key resources table, are available from the lead contact upon request.

Data and code availability

Genotype information for cell lines used are publicly available through the HapMap Project (https://ftp.ncbi.nlm.nih.gov/hapmap/; r28) and the 1000 Genomes Project (https://ftp.1000genomes.ebi.ac.uk/vol1/ftp/; v.20130520). Phenotype information is provided in Table S1. Genome-wide association summary statistics have been deposited at the Duke Research Data Repository (Duke Research Data Repository: https://doi.org/10.7924/r43n2d008). Raw Imaging, raw western blot images, and KO cell sequencing files are available at Mendeley Data (Mendeley Data: https://doi.org/10.17632/hs467zj2cw.1).

ACKNOWLEDGMENTS

We thank the investigators and individuals from diverse populations genotyped as part of the 1000 Genomes Project who have made their LCLs available through the Coriell Institute. We thank members of the Ko lab for useful discussion. R.M.K., S.S., T.D., L.W., and D.C.K. were supported by NIH grant no. R01AI118903. R.M.K. and T.D. were supported by TriCEM Graduate Student Fellowships. R.M.K. was supported by a Duke Precision Genomics Center student pilot grant. M.A.L. and N.M.R.-V. were supported by NIH grant no. R01CA140337.

AUTHOR CONTRIBUTIONS

Conceptualization, R.M.K., S.I.M., T.J.W., and D.C.K. Formal analysis, R.M.K., S.S., T.D., L.W., and D.C.K. Investigation, R.M.K., S.S., T.D., L.W., and D.C.K. Funding acquisition, R.M.K. and D.C.K. Supervision, R.M.K., N.M.R.-V., M.A.L., S.I.M., and D.C.K. Resources, R.M.K., S.S., N.M.R.-V., M.A.L., and D.C.K. Writing – original draft, R.M.K., S.S., T.D., L.W., and D.C.K. Writing – review & editing, all authors.

DECLARATION OF INTERESTS

The authors declare no competing interests.

STAR★METHODS

Detailed methods are provided in the online version of this paper and include the following:

- KEY RESOURCES TABLE
- EXPERIMENTAL MODEL AND STUDY PARTICIPANT DETAILS
  - Human cell culture
  - Bacterial cell culture
- METHOD DETAILS
  - Human cell infection assays
  - Cas9-RNP based editing of LCLs
  - HeLa overexpression transfection
  - HeLa microscopy
  - Quantification of Syk and FCRL3 clustering by microscopy
  - Construction of FCRL3 mutants
  - Purification of FCRL5 extracellular domain and *Y. pestis* binding assay
  - Western blot
  - Flow cytometry of PBMCs
- QUANTIFICATION AND STATISTICAL ANALYSIS
  - Genome-wide association
  - Phenotype- and SNP-based heritability analysis
  - Colocalization analysis of Hi-HOST *Y. pestis* phenotype and BioBank Japan chronic hepatitis C GWAS
  - Descriptive statistics and visualization

SUPPLEMENTAL INFORMATION

Supplemental information can be found online at https://doi.org/10.1016/j.xgen.2025.100917.

Received: December 2, 2024
Revised: March 31, 2025
Accepted: May 13, 2025
Published: June 9, 2025

REFERENCES

1. Fumagalli, M., and Sironi, M. (2014). Human genome variability, natural selection and infectious diseases. Curr. Opin. Immunol. *30*, 9–16. https://doi.org/10.1016/j.coi.2014.05.001.
2. Pittman, K.J., Glover, L.C., Wang, L., and Ko, D.C. (2016). The Legacy of Past Pandemics: Common Human Mutations That Protect against Infectious Disease. PLoS Pathog. *12*, e1005680. https://doi.org/10.1371/journal.ppat.1005680.

3. Barbieri, R., Signoli, M., Cheve, D., Costedoat, C., Tzortzis, S., Aboud-haram, G., Raoult, D., and Drancourt, M. (2020). Yersinia pestis: the Natural History of Plague. Clin. Microbiol. Rev. *34*, e00044-19. https://doi.org/10.1128/CMR.00044-19.

4. Benedictow, O.J. (2004). The Black Death, 1346-1353: The Complete History (Boydell Press).

5. Salam, A.P., Rojek, A., Cai, E., Raberahona, M., and Horby, P. (2020). Deaths Associated with Pneumonic Plague, 1946-2017. Emerg. Infect. Dis. *26*, 2432–2434. https://doi.org/10.3201/eid2610.191270.

6. Yang, K., He, Y., Park, C.G., Kang, Y.S., Zhang, P., Han, Y., Cui, Y., Bulgheresi, S., Anisimov, A.P., Dentovskaya, S.V., et al. (2019). Yersinia pestis Interacts With SIGNR1 (CD209b) for Promoting Host Dissemination and Infection. Front. Immunol. *10*, 96. https://doi.org/10.3389/fimmu.2019.00096.

7. St John, A.L., Ang, W.X.G., Huang, M.N., Kunder, C.A., Chan, E.W., Gunn, M.D., and Abraham, S.N. (2014). S1P-Dependent trafficking of intracellular yersinia pestis through lymph nodes establishes Buboes and systemic infection. Immunity *41*, 440–450. https://doi.org/10.1016/j.immuni.2014.07.013.

8. Connor, M.G., Pulsifer, A.R., Chung, D., Rouchka, E.C., Ceresa, B.K., and Lawrenz, M.B. (2018). Yersinia pestis Targets the Host Endosome Recycling Pathway during the Biogenesis of the Yersinia-Containing Vacuole To Avoid Killing by Macrophages. mBio *9*, e01800-17. https://doi.org/10.1128/mBio.01800-17.

9. Osei-Owusu, P., Charlton, T.M., Kim, H.K., Missiakas, D., and Schneewind, O. (2019). FPR1 is the plague receptor on host immune cells. Nature *574*, 57–62. https://doi.org/10.1038/s41586-019-1570-z.

10. Klunk, J., Vilgalys, T.P., Demeure, C.E., Cheng, X., Shiratori, M., Madej, J., Beau, R., Elli, D., Patino, M.I., Redfern, R., et al. (2022). Evolution of immune genes is associated with the Black Death. Nature *611*, 312–319. https://doi.org/10.1038/s41586-022-05349-x.

11. Immel, A., Key, F.M., Szolek, A., Barquera, R., Robinson, M.K., Harrison, G.F., Palmer, W.H., Spyrou, M.A., Susat, J., Krause-Kyora, B., et al. (2021). Analysis of Genomic DNA from Medieval Plague Victims Suggests Long-Term Effect of Yersinia pestis on Human Immunity Genes. Mol. Biol. Evol. *38*, 4059–4076. https://doi.org/10.1093/molbev/msab147.

12. Gibbs, K.D., Schott, B.H., and Ko, D.C. (2022). The Awesome Power of Human Genetics of Infectious Disease. Annu. Rev. Genet. *56*, 41–62.

13. Hill, A.V.S. (2012). Evolution, revolution and heresy in the genetics of infectious disease susceptibility. Philos. Trans. R. Soc. Lond. B Biol. Sci. *367*, 840–849. https://doi.org/10.1098/rstb.2011.0275.

14. Dean, M., Carrington, M., Winkler, C., Huttley, G.A., Smith, M.W., Allikmets, R., Goedert, J.J., Buchbinder, S.P., Vittinghoff, E., Gomperts, E., et al. (1996). Genetic restriction of HIV-1 infection and progression to AIDS by a deletion allele of the CKR5 structural gene. Hemophilia Growth and Development Study, Multicenter AIDS Cohort Study, Multicenter Hemophilia Cohort Study, San Francisco City Cohort, ALIVE Study. Science *273*, 1856–1862.

15. Liu, R., Paxton, W.A., Choe, S., Ceradini, D., Martin, S.R., Horuk, R., MacDonald, M.E., Stuhlmann, H., Koup, R.A., and Landau, N.R. (1996). Homozygous defect in HIV-1 coreceptor accounts for resistance of some multiply-exposed individuals to HIV-1 infection. Cell *86*, 367–377.

16. Samson, M., Libert, F., Doranz, B.J., Rucker, J., Liesnard, C., Farber, C. M., Saragosti, S., Lapoumeroulie, C., Cognaux, J., Forceille, C., et al. (1996). Resistance to HIV-1 infection in caucasian individuals bearing mutant alleles of the CCR-5 chemokine receptor gene. Nature *382*, 722–725. https://doi.org/10.1038/382722a0.

17. Jensen, B.E.O., Knops, E., Cords, L., Lübke, N., Salgado, M., Busman-Sahay, K., Estes, J.D., Huyveneers, L.E.P., Perdomo-Celis, F., Wittner, M., et al. (2023). In-depth virological and immunological characterization of HIV-1 cure after CCR5Delta32/Delta32 allogeneic hematopoietic stem cell transplantation. Nat. Med. *29*, 583–587. https://doi.org/10.1038/s41591-023-02213-x.

18. Hutter, G., Nowak, D., Mossner, M., Ganepola, S., Mussig, A., Allers, K., Schneider, T., Hofmann, J., Kucherer, C., Blau, O., et al. (2009). Long-term control of HIV by CCR5 Delta32/Delta32 stem-cell transplantation. N. Engl. J. Med. *360*, 692–698. https://doi.org/10.1056/NEJMoa0802905.

19. Lindesmith, L., Moe, C., Marionneau, S., Ruvoen, N., Jiang, X., Lindblad, L., Stewart, P., LePendu, J., and Baric, R. (2003). Human susceptibility and resistance to Norwalk virus infection. Nat. Med. *9*, 548–553. https://doi.org/10.1038/nm860.

20. Isberg, R.R., and Leong, J.M. (1990). Multiple beta 1 chain integrins are receptors for invasin, a protein that promotes bacterial penetration into mammalian cells. Cell *60*, 861–871. https://doi.org/10.1016/0092-8674(90)90099-z.

21. Isberg, R.R., Voorhis, D.L., and Falkow, S. (1987). Identification of invasin: a protein that allows enteric bacteria to penetrate cultured mammalian cells. Cell *50*, 769–778. https://doi.org/10.1016/0092-8674(87)90335-7.

22. Parkhill, J., Wren, B.W., Thomson, N.R., Titball, R.W., Holden, M.T., Prentice, M.B., Sebaihia, M., James, K.D., Churcher, C., Mungall, K.L., et al. (2001). Genome sequence of Yersinia pestis, the causative agent of plague. Nature *413*, 523–527. https://doi.org/10.1038/35097083.

23. Zhang, S.S., Park, C.G., Zhang, P., Bartra, S.S., Plano, G.V., Klena, J.D., Skurnik, M., Hinnebusch, B.J., and Chen, T. (2008). Plasminogen activator Pla of Yersinia pestis utilizes murine DEC-205 (CD205) as a receptor to promote dissemination. J. Biol. Chem. *283*, 31511–31521. https://doi.org/10.1074/jbc.M804646200.

24. Arifuzzaman, M., Ang, W.X.G., Choi, H.W., Nilles, M.L., St John, A.L., and Abraham, S.N. (2018). Necroptosis of infiltrated macrophages drives Yersinia pestis dispersal within buboes. JCI Insight *3*, e122188. https://doi.org/10.1172/jci.insight.122188.

25. Li, B., and Yang, R. (2008). Interaction between Yersinia pestis and the host immune system. Infect. Immun. *76*, 1804–1811. https://doi.org/10.1128/IAI.01517-07.

26. Zhao, Y., Wang, T., Liu, Z., Ke, Y., Li, R., Chen, H., You, Y., Wu, G., Cao, S., Du, Z., et al. (2023). Single-cell transcriptomics of immune cells in lymph nodes reveals their composition and alterations in functional dynamics during the early stages of bubonic plague. Sci. China Life Sci. *66*, 110–126. https://doi.org/10.1007/s11427-021-2119-5.

27. Gibbs, K.D., Wang, L., Yang, Z., Anderson, C.E., Bourgeois, J.S., Cao, Y., Gaggioli, M.R., Biel, M., Puertollano, R., Chen, C.C., and Ko, D.C. (2023). Human variation impacting MCOLN2 restricts Salmonella Typhi replication by magnesium deprivation. Cell Genom. *3*, 100290. https://doi.org/10.1016/j.xgen.2023.100290.

28. Ko, D.C., Shukla, K.P., Fong, C., Wasnick, M., Brittnacher, M.J., Wurfel, M.M., Holden, T.D., O'Keefe, G.E., Van Yserloo, B., Akey, J.M., and Miller, S.I. (2009). A genome-wide in vitro bacterial-infection screen reveals human variation in the host response associated with inflammatory disease. Am. J. Hum. Genet. *85*, 214–227. https://doi.org/10.1016/j.ajhg.2009.07.012.

29. Wang, L., Pittman, K.J., Barker, J.R., Salinas, R.E., Stanaway, I.B., Williams, G.D., Carroll, R.J., Balmat, T., Ingham, A., Gopalakrishnan, A.M., et al. (2018). An Atlas of Genetic Variation Linking Pathogen-Induced Cellular Traits to Human Disease. Cell Host Microbe *24*, 308–323.e6. https://doi.org/10.1016/j.chom.2018.07.007.

30. Janssen, W.A., Lawton, W.D., Fukui, G.M., and Surgalla, M.J. (1963). The Pathogenesis of Plague. I. A Study of the Correlation between Virulence and Relative Phagocytosis Resistance of Some Strains of Pasteurella Pestis. J. Infect. Dis. *113*, 139–143. https://doi.org/10.1093/infdis/113.2.139.

31. Fallman, M., Deleuil, F., and McGee, K. (2002). Resistance to phagocytosis by Yersinia. Int J Med Microbiol *291*, 501–509. https://doi.org/10.1078/1438-4221-00159.

32. Burrows, T.W., and Bacon, G.A. (1956). The basis of virulence in Pasteurella pestis: the development of resistance to phagocytosis in vitro. Br. J. Exp. Pathol. *37*, 286–299.

33. Spinner, J.L., Cundiff, J.A., and Kobayashi, S.D. (2008). Yersinia pestis type III secretion system-dependent inhibition of human polymorphonuclear leukocyte function. Infect. Immun. *76*, 3754–3760. https://doi.org/10.1128/IAI.00385-08.

34. International HapMap Consortium (2005). A haplotype map of the human genome. Nature *437*, 1299–1320. https://doi.org/10.1038/nature04226.

35. Frazer, K.A., Ballinger, D.G., Cox, D.R., Hinds, D.A., Stuve, L.L., Gibbs, R. A., Belmont, J.W., Boudreau, A., Hardenbol, P., et al.; International HapMap Consortium (2007). A second generation human haplotype map of over 3.1 million SNPs. Nature *449*, 851–861. https://doi.org/10.1038/nature06258.

36. 1000 Genomes Project Consortium; Abecasis, G.R., Auton, A., Brooks, L.D., DePristo, M.A., Durbin, R.M., Handsaker, R.E., Kang, H.M., Marth, G.T., and McVean, G.A. (2012). An integrated map of genetic variation from 1,092 human genomes. Nature *491*, 56–65. https://doi.org/10.1038/nature11632.

37. 1000 Genomes Project Consortium; Auton, A., Brooks, L.D., Durbin, R. M., Garrison, E.P., Kang, H.M., Korbel, J.O., Marchini, J.L., McCarthy, S., McVean, G.A., and Abecasis, G.R. (2015). A global reference for human genetic variation. Nature *526*, 68–74. https://doi.org/10.1038/nature15393.

38. Lewis, M.J., and Wang, S. (2025). locuszoomr: an R package for visualizing publication-ready regional gene locus plots. Bioinform. Adv. *5*, vbaf006. https://doi.org/10.1093/bioadv/vbaf006.

39. Straley, S.C., and Harmon, P.A. (1984). Growth in mouse peritoneal macrophages of Yersinia pestis lacking established virulence determinants. Infect. Immun. *45*, 649–654. https://doi.org/10.1128/iai.45.3.649-654.1984.

40. Charnetzky, W.T., and Shuford, W.W. (1985). Survival and growth of Yersinia pestis within macrophages and an effect of the loss of the 47-megadalton plasmid on growth in macrophages. Infect. Immun. *47*, 234–241. https://doi.org/10.1128/iai.47.1.234-241.1985.

41. Purcell, S., Neale, B., Todd-Brown, K., Thomas, L., Ferreira, M.A.R., Bender, D., Maller, J., Sklar, P., de Bakker, P.I.W., Daly, M.J., and Sham, P.C. (2007). PLINK: a tool set for whole-genome association and population-based linkage analyses. Am. J. Hum. Genet. *81*, 559–575. https://doi.org/10.1086/519795.

42. Purcell, S., Sham, P., and Daly, M.J. (2005). Parental phenotypes in family-based association analysis. Am. J. Hum. Genet. *76*, 249–259. https://doi.org/10.1086/427886.

43. Schork, A.J., Thompson, W.K., Pham, P., Torkamani, A., Roddey, J.C., Sullivan, P.F., Kelsoe, J.R., O'Donovan, M.C., Furberg, H., et al.; Tobacco and Genetics Consortium (2013). All SNPs are not created equal: genome-wide association studies reveal a consistent pattern of enrichment among functionally annotated SNPs. PLoS Genet. *9*, e1003449. https://doi.org/10.1371/journal.pgen.1003449.

44. Davis, R.S. (2007). Fc receptor-like molecules. Annu. Rev. Immunol. *25*, 525–560. https://doi.org/10.1146/annurev.immunol.25.022106.141541.

45. Tolnay, M. (2022). Lymphocytes sense antibodies through human FCRL proteins: Emerging roles in mucosal immunity. J. Leukoc. Biol. *111*, 477–487. https://doi.org/10.1002/JLB.4RU0221-102RR.

46. Agarwal, S., Kraus, Z., Dement-Brown, J., Alabi, O., Starost, K., and Tolnay, M. (2020). Human Fc Receptor-like 3 Inhibits Regulatory T Cell Function and Binds Secretory IgA. Cell Rep. *30*, 1292–1299.e3. https://doi.org/10.1016/j.celrep.2019.12.099.

47. Wilson, T.J., Fuchs, A., and Colonna, M. (2012). Cutting edge: human FcRL4 and FcRL5 are receptors for IgA and IgG. J. Immunol. *188*, 4741–4745. https://doi.org/10.4049/jimmunol.1102651.

48. Zhao, X., Zhang, G., Liu, S., Chen, X., Peng, R., Dai, L., Qu, X., Li, S., Song, H., Gao, Z., et al. (2019). Human Neonatal Fc Receptor Is the Cellular Uncoating Receptor for Enterovirus B. Cell *177*, 1553–1565. e16. https://doi.org/10.1016/j.cell.2019.04.035.

49. Maidji, E., McDonagh, S., Genbacev, O., Tabata, T., and Pereira, L. (2006). Maternal antibodies enhance or prevent cytomegalovirus infection in the placenta by neonatal Fc receptor-mediated transcytosis. Am. J. Pathol. *168*, 1210–1226. https://doi.org/10.2353/ajpath.2006.050482.

50. Laassili, C., Ben El Hend, F., Benzidane, R., Oumeslakht, L., Aziz, A.I., El Fatimy, R., Bensussan, A., and Ben Mkaddem, S. (2023). Fc receptors act as innate immune receptors during infection? Front. Immunol. *14*, 1188497. https://doi.org/10.3389/fimmu.2023.1188497.

51. Indik, Z.K., Park, J.G., Hunter, S., and Schreiber, A.D. (1995). Structure/function relationships of Fc gamma receptors in phagocytosis. Semin. Immunol. *7*, 45–54. https://doi.org/10.1016/1044-5323(95)90007-1.

52. Charles A Janeway, J., Paul, T., Walport, M., and Shlomchik, M.J. (2001). The Destruction of Antibody-Coated Pathogens via Fc Receptors, 5th Edition (Garland Science).

53. Tay, M.Z., Wiehe, K., and Pollara, J. (2019). Antibody-Dependent Cellular Phagocytosis in Antiviral Immune Responses. Front. Immunol. *10*, 332. https://doi.org/10.3389/fimmu.2019.00332.

54. Indik, Z.K., Park, J.G., Pan, X.Q., and Schreiber, A.D. (1995). Induction of phagocytosis by a protein tyrosine kinase. Blood *85*, 1175–1180.

55. Xu, M.J., Zhao, R., Cao, H., and Zhao, Z.J. (2002). SPAP2, an Ig family receptor containing both ITIMs and ITAMs. Biochem. Biophys. Res. Commun. *293*, 1037–1046. https://doi.org/10.1016/S0006-291X(02)00332-7.

56. Getahun, A., and Cambier, J.C. (2015). Of ITIMs, ITAMs, and ITAMis: revisiting immunoglobulin Fc receptor signaling. Immunol. Rev. *268*, 66–73. https://doi.org/10.1111/imr.12336.

57. Crowley, M.T., Costello, P.S., Fitzer-Attas, C.J., Turner, M., Meng, F., Lowell, C., Tybulewicz, V.L., and DeFranco, A.L. (1997). A critical role for Syk in signal transduction and phagocytosis mediated by Fcgamma receptors on macrophages. J. Exp. Med. *186*, 1027–1039. https://doi.org/10.1084/jem.186.7.1027.

58. Kochi, Y., Myouzen, K., Yamada, R., Suzuki, A., Kurosaki, T., Nakamura, Y., and Yamamoto, K. (2009). FCRL3, an autoimmune susceptibility gene, has inhibitory potential on B-cell receptor-mediated signaling. J. Immunol. *183*, 5502–5510. https://doi.org/10.4049/jimmunol.0901982.

59. Bauer, B., and Steinle, A. (2017). HemITAM: A single tyrosine motif that packs a punch. Sci. Signal. *10*, eaan3676. https://doi.org/10.1126/scisignal.aan3676.

60. Uribe-Querol, E., and Rosales, C. (2020). Phagocytosis: Our Current Understanding of a Universal Biological Process. Front. Immunol. *11*, 1066. https://doi.org/10.3389/fimmu.2020.01066.

61. Herre, J., Marshall, A.S.J., Caron, E., Edwards, A.D., Williams, D.L., Schweighoffer, E., Tybulewicz, V., Reis e Sousa, C., Gordon, S., and Brown, G.D. (2004). Dectin-1 uses novel mechanisms for yeast phagocytosis in macrophages. Blood *104*, 4038–4045. https://doi.org/10.1182/blood-2004-03-1140.

62. Zhang, P., Schwartz, O., Pantelic, M., Li, G., Knazze, Q., Nobile, C., Radovich, M., He, J., Hong, S.C., Klena, J., and Chen, T. (2006). DC-SIGN (CD209) recognition of Neisseria gonorrhoeae is circumvented by lipooligosaccharide variation. J. Leukoc. Biol. *79*, 731–738. https://doi.org/10.1189/jlb.0405184.

63. Du, Y., Rosqvist, R., and Forsberg, A. (2002). Role of fraction 1 antigen of Yersinia pestis in inhibition of phagocytosis. Infect. Immun. *70*, 1453–1460. https://doi.org/10.1128/IAI.70.3.1453-1460.2002.

64. Bonglack, E.N., Messinger, J.E., Cable, J.M., Ch'ng, J., Parnell, K.M., Reinoso-Vizcaíno, N.M., Barry, A.P., Russell, V.S., Dave, S.S., Christofk, H.R., and Luftig, M.A. (2021). Monocarboxylate transporter antagonism reveals metabolic vulnerabilities of viral-driven lymphomas. Proc. Natl.

Acad. Sci. USA *118*, e2022495118. https://doi.org/10.1073/pnas.2022495118.

65. SoRelle, E.D., Reinoso-Vizcaino, N.M., Dai, J., Barry, A.P., Chan, C., and Luftig, M.A. (2023). Epstein-Barr virus evades restrictive host chromatin closure by subverting B cell activation and germinal center regulatory loci. Cell Rep. *42*, 112958. https://doi.org/10.1016/j.celrep.2023.112958.
66. Perry, R.D., and Fetherston, J.D. (1997). Yersinia pestis–etiologic agent of plague. Clin. Microbiol. Rev. *10*, 35–66. https://doi.org/10.1128/CMR.10.1.35.
67. Achtman, M., Zurth, K., Morelli, G., Torrea, G., Guiyoule, A., and Carniel, E. (1999). Yersinia pestis, the cause of plague, is a recently emerged clone of Yersinia pseudotuberculosis. Proc. Natl. Acad. Sci. USA *96*, 14043–14048. https://doi.org/10.1073/pnas.96.24.14043.
68. Brubaker, R.R. (1991). Factors promoting acute and chronic diseases caused by yersiniae. Clin. Microbiol. Rev. *4*, 309–324. https://doi.org/10.1128/CMR.4.3.309.
69. Califf, K.J., Keim, P.S., Wagner, D.M., and Sahl, J.W. (2015). Redefining the differences in gene content between Yersinia pestis and Yersinia pseudotuberculosis using large-scale comparative genomics. Microb. Genom. *1*, e000028. https://doi.org/10.1099/mgen.0.000028.
70. Chain, P.S.G., Carniel, E., Larimer, F.W., Lamerdin, J., Stoutland, P.O., Regala, W.M., Georgescu, A.M., Vergez, L.M., Land, M.L., Motin, V.L., et al. (2004). Insights into the evolution of Yersinia pestis through whole-genome comparison with Yersinia pseudotuberculosis. Proc. Natl. Acad. Sci. USA *101*, 13826–13831. https://doi.org/10.1073/pnas.0404012101.
71. Wilson, T.J., Gilfillan, S., and Colonna, M. (2010). Fc receptor-like A associates with intracellular IgG and IgM but is dispensable for antigen-specific immune responses. J. Immunol. *185*, 2960–2967. https://doi.org/10.4049/jimmunol.1001428.
72. Li, M., and Yu, Y. (2021). Innate immune receptor clustering and its role in immune regulation. J. Cell Sci. *134*, jcs249318. https://doi.org/10.1242/jcs.249318.
73. Li, F.J., Won, W.J., Becker, E.J., Jr., Easlick, J.L., Tabengwa, E.M., Li, R., Shakhmatov, M., Honjo, K., Burrows, P.D., and Davis, R.S. (2014). Emerging roles for the FCRL family members in lymphocyte biology and disease. Curr. Top. Microbiol. Immunol. *382*, 29–50. https://doi.org/10.1007/978-3-319-07911-0_2.
74. Wojtowicz, W.M., Vielmetter, J., Fernandes, R.A., Siepe, D.H., Eastman, C.L., Chisholm, G.B., Cox, S., Klock, H., Anderson, P.W., Rue, S.M., et al. (2020). A Human IgSF Cell-Surface Interactome Reveals a Complex Network of Protein-Protein Interactions. Cell *182*, 1027–1043.e17. https://doi.org/10.1016/j.cell.2020.07.025.
75. Nagai, A., Hirata, M., Kamatani, Y., Muto, K., Matsuda, K., Kiyohara, Y., Ninomiya, T., Tamakoshi, A., Yamagata, Z., Mushiroda, T., et al. (2017). Overview of the BioBank Japan Project: Study design and profile. J. Epidemiol. *27*, S2–S8. https://doi.org/10.1016/j.je.2016.12.005.
76. Sakaue, S., Kanai, M., Tanigawa, Y., Karjalainen, J., Kurki, M., Koshiba, S., Narita, A., Konuma, T., Yamamoto, K., Akiyama, M., et al. (2021). A cross-population atlas of genetic associations for 220 human phenotypes. Nat. Genet. *53*, 1415–1424. https://doi.org/10.1038/s41588-021-00931-x.
77. Giambartolomei, C., Vukcevic, D., Schadt, E.E., Franke, L., Hingorani, A.D., Wallace, C., and Plagnol, V. (2014). Bayesian test for colocalisation between pairs of genetic association studies using summary statistics. PLoS Genet. *10*, e1004383. https://doi.org/10.1371/journal.pgen.1004383.
78. Ke, Y., Chen, Z., and Yang, R. (2013). Yersinia pestis: mechanisms of entry into and resistance to the host cell. Front. Cell. Infect. Microbiol. *3*, 106. https://doi.org/10.3389/fcimb.2013.00106.
79. Debes, G.F., and McGettigan, S.E. (2019). Skin-Associated B Cells in Health and Inflammation. J. Immunol. *202*, 1659–1666. https://doi.org/10.4049/jimmunol.1801211.
80. Yother, J., Chamness, T.W., and Goguen, J.D. (1986). Temperature-controlled plasmid regulon associated with low calcium response in Yersinia pestis. J. Bacteriol. *165*, 443–447. https://doi.org/10.1128/jb.165.2.443-447.1986.
81. Hsiao, T.L., and Vitkup, D. (2008). Role of duplicate genes in robustness against deleterious human mutations. PLoS Genet. *4*, e1000014. https://doi.org/10.1371/journal.pgen.1000014.
82. de Tymowski, C., Heming, N., Correia, M.D.T., Abbad, L., Chavarot, N., Le Stang, M.B., Flament, H., Bex, J., Boedec, E., Bounaix, C., et al. (2019). CD89 Is a Potent Innate Receptor for Bacteria and Mediates Host Protection from Sepsis. Cell Rep. *27*, 762–775.e5. https://doi.org/10.1016/j.celrep.2019.03.062.
83. Pinheiro da Silva, F., Aloulou, M., Skurnik, D., Benhamou, M., Andremont, A., Velasco, I.T., Chiamolera, M., Verbeek, J.S., Launay, P., and Monteiro, R.C. (2007). CD16 promotes Escherichia coli sepsis through an FcR gamma inhibitory pathway that prevents phagocytosis and facilitates inflammation. Nat. Med. *13*, 1368–1374. https://doi.org/10.1038/nm1665.
84. Field, Y., Boyle, E.A., Telis, N., Gao, Z., Gaulton, K.J., Golan, D., Yengo, L., Rocheleau, G., Froguel, P., McCarthy, M.I., and Pritchard, J.K. (2016). Detection of human adaptation during the past 2000 years. Science *354*, 760–764. https://doi.org/10.1126/science.aag0776.
85. Taliun, D., Harris, D.N., Kessler, M.D., Carlson, J., Szpiech, Z.A., Torres, R., Taliun, S.A.G., Corvelo, A., Gogarten, S.M., Kang, H.M., et al. (2021). Sequencing of 53,831 diverse genomes from the NHLBI TOPMed Program. Nature *590*, 290–299. https://doi.org/10.1038/s41586-021-03205-y.
86. Chen, C.L., Huang, J.Y., Wang, C.H., Tahara, S.M., Zhou, L., Kondo, Y., Schechter, J., Su, L., Lai, M.M.C., Wakita, T., et al. (2017). Hepatitis C virus has a genetically determined lymphotropism through co-receptor B7.2. Nat. Commun. *8*, 13882. https://doi.org/10.1038/ncomms13882.
87. Desombere, I., Van Houtte, F., Farhoudi, A., Verhoye, L., Buysschaert, C., Gijbels, Y., Couvent, S., Swinnen, W., Van Vlierberghe, H., Elewaut, A., et al. (2021). A Role for B Cells to Transmit Hepatitis C Virus Infection. Front. Immunol. *12*, 775098. https://doi.org/10.3389/fimmu.2021.775098.
88. GTEx Consortium (2020). The GTEx Consortium atlas of genetic regulatory effects across human tissues. Science (New York, N.Y.) *369*, 1318–1330. https://doi.org/10.1126/science.aaz1776.
89. Sun, B.B., Maranville, J.C., Peters, J.E., Stacey, D., Staley, J.R., Blackshaw, J., Burgess, S., Jiang, T., Paige, E., Surendran, P., et al. (2018). Genomic atlas of the human plasma proteome. Nature *558*, 73–79. https://doi.org/10.1038/s41586-018-0175-2.
90. Suhre, K., Arnold, M., Bhagwat, A.M., Cotton, R.J., Engelke, R., Raffler, J., Sarwath, H., Thareja, G., Wahl, A., DeLisle, R.K., et al. (2017). Connecting genetic risk to disease end points through the human blood plasma proteome. Nat. Commun. *8*, 14357. https://doi.org/10.1038/ncomms14357.
91. Zhao, S.X., Xue, L.Q., Liu, W., Gu, Z.H., Pan, C.M., Yang, S.Y., Zhan, M., Wang, H.N., Liang, J., Gao, G.Q., et al. (2013). Robust evidence for five new Graves' disease risk loci from a staged genome-wide association analysis. Hum. Mol. Genet. *22*, 3347–3362. https://doi.org/10.1093/hmg/ddt183.
92. Ha, E., Bae, S.C., and Kim, K. (2021). Large-scale meta-analysis across East Asian and European populations updated genetic architecture and variant-driven biology of rheumatoid arthritis, identifying 11 novel susceptibility loci. Ann. Rheum. Dis. *80*, 558–565. https://doi.org/10.1136/annrheumdis-2020-219065.
93. Kim, K., Bang, S.Y., Lee, H.S., Cho, S.K., Choi, C.B., Sung, Y.K., Kim, T.H., Jun, J.B., Yoo, D.H., Kang, Y.M., et al. (2015). High-density genotyping of immune loci in Koreans and Europeans identifies eight new rheumatoid arthritis risk loci. Ann. Rheum. Dis. *74*, e13. https://doi.org/10.1136/annrheumdis-2013-204749.

94. Kochi, Y., Yamada, R., Suzuki, A., Harley, J.B., Shirasawa, S., Sawada, T., Bae, S.C., Tokuhiro, S., Chang, X., Sekine, A., et al. (2005). A functional variant in FCRL3, encoding Fc receptor-like 3, is associated with rheumatoid arthritis and several autoimmunities. Nat. Genet. *37*, 478–485. https://doi.org/10.1038/ng1540.

95. Sawcer, S., Hellenthal, G., Pirinen, M., Spencer, C.C.A., Patsopoulos, N. A., Moutsianas, L., Dilthey, A., Su, Z., et al.; International Multiple Sclerosis Genetics Consortium; Wellcome Trust Case Control Consortium 2 (2011). Genetic risk and a primary role for cell-mediated immune mechanisms in multiple sclerosis. Nature *476*, 214–219. https://doi.org/10.1038/nature10251.

96. International Multiple Sclerosis Genetics, C., Patsopoulos, N.A., Baranzini, S.E., Santaniello, A., Shoostari, P., Cotsapas, C., Wong, G., Beecham, A.H., James, T., Replogle, J., et al. (2019). Multiple sclerosis genomic map implicates peripheral immune cells and microglia in susceptibility. Science *365*, eaav7188. https://doi.org/10.1126/science.aav7188.

97. Pujol, C., and Bliska, J.B. (2003). The ability to replicate in macrophages is conserved between Yersinia pestis and Yersinia pseudotuberculosis. Infect. Immun. *71*, 5892–5899.

98. Chang, C.C., Chow, C.C., Tellier, L.C., Vattikuti, S., Purcell, S.M., and Lee, J.J. (2015). Second-generation PLINK: rising to the challenge of larger and richer datasets. GigaScience *4*, 7. https://doi.org/10.1186/s13742-015-0047-8.

99. Schneider, C.A., Rasband, W.S., and Eliceiri, K.W. (2012). NIH Image to ImageJ: 25 years of image analysis. Nat. Methods *9*, 671–675.

100. Stoffel, M.A., Nakagawa, S., and Schielzeth, H. (2017). rptR: repeatability estimation and variance decomposition by generalized linear mixed-effects models. Methods Ecol. Evol. *8*, 1639–1644. https://doi.org/10.1111/2041-210x.12797.

101. Paria, S.S., Rahman, S.R., and Adhikari, K. (2022). fastman: A fast algorithm for visualizing GWAS results using Manhattan and Q-Q plots. Preprint at bioRxiv. https://doi.org/10.1101/2022.04.19.488738.

102. 1000 Genomes Project Consortium; Abecasis, G.R., Altshuler, D., Auton, A., Brooks, L.D., Durbin, R.M., Gibbs, R.A., Hurles, M.E., and McVean, G.A. (2010). A map of human genome variation from population-scale sequencing. Nature *467*, 1061–1073. https://doi.org/10.1038/nature09534.

103. Yang, J., Lee, S.H., Goddard, M.E., and Visscher, P.M. (2011). GCTA: a tool for genome-wide complex trait analysis. Am. J. Hum. Genet. *88*, 76–82. https://doi.org/10.1016/j.ajhg.2010.11.011.

104. Team, R. (2020). RStudio: Integrated Development for R.

105. Pruim, R.J., Welch, R.P., Sanna, S., Teslovich, T.M., Chines, P.S., Gliedt, T.P., Boehnke, M., Abecasis, G.R., and Willer, C.J. (2010). LocusZoom: regional visualization of genome-wide association scan results. Bioinformatics *26*, 2336–2337. https://doi.org/10.1093/bioinformatics/btq419.

STAR★METHODS

KEY RESOURCES TABLE

REAGENT or RESOURCE	SOURCE	IDENTIFIER
Antibodies		
FcRH3 Antibody (C-2)	Santa Cruz	Cat#sc-365706; RRID: AB_10848450
Polyclonal Anti-Yersinia pestis F1-Antigen (antiserum, Goat)	BEI Resources	NR-31024
DYKDDDDK Tag antibody (D6W5B)	Cell Signaling	Cat#14793; RRID: AB_2572291
Monoclonal ANTI-FLAG M2	Sigma-Aldrich	Cat#F3165; RRID: AB_259529
CD31 (PECAM-1) (89C2) Mouse mAb	Cell Signaling	Cat#3528; RRID: AB_2160882
Myc-Tag (71D10) Rabbit mAb	Cell Signaling	Cat#2278; RRID: AB_490778
Syk (4D10) Mouse mAb	Cell Signaling	Cat#80460; RRID: AB_2799953
Avi Tag Monoclonal Antibody (1D11D10)	ThermoFisher	Cat#A01738-40
PE anti-human CD46 Antibody (TRA-2-10)	BioLegend	Cat#352401; RRID: AB_10895912
c-MYC (9E 10)	DSHB	Cat#9E 10-s, RRID: AB_2266850
LAMP1 (H4A3)	DSHB	Cat#H4A3, RRID: AB_2296838
PE anti-human CD3 (UCHT1)	BD Biosciences	Cat#561808; RRID: AB_10893403
CD19 Monoclonal Antibody (HIB19), PE-Cyanine5	eBioscience	Cat#15-0199-71
Dynabeads His-Tag Isolation and Pulldown	ThermoFisher	Cat#10103D
Bacterial and virus strains		
Yersinia pestis KIM6+ +p67GFP3.1	This paper	DCK41
Yersinia pestis KIM5 +p67GFP3.1	This paper	DCK1195
Yersinia pseudotuberculosis IP32935 + p67GFP3.1	This paper	DCK27
Biological samples		
Human peripheral blood mononuclear cells	Lonza	Cat#CC-2704; Lot: 24TL249158
Human peripheral blood mononuclear cells	Lonza	Cat#CC-2704; Lot: 24TL312076
Chemicals, peptides, and recombinant proteins		
7-aminoactinomycin D (7AAD)	Enzo	Cat#ALX-380-283
Octyl β-D-glucopyranoside	Sigma	Cat#O8001
Gentamicin Sulfate	VWR	Cat#45000-634
Isopropyl ß-D-1-thiogalactopyranoside (IPTG)	ThermoFisher	Cat#15529–019
TrueCut^{TM} Cas9 Protein v2	ThermoFisher	Cat#A36498
Syk Inhibitor IV, BAY 61–3606 HCl (CAS 732983-37-8)	Santa Cruz	Cat#sc-202351
NucBlue Live ReadyProbes Reagent (Hoechst 33342)	ThermoFisher	Cat#R37605
DAPI (4′,6-diamidino-2-phenylindole, dihydrochloride)	ThermoFisher	Cat#D1306
Fluoromount-G Mounting Medium	ThermoFisher	Cat#00-4958-02
mini-cOmplete protease inhibitor tablet	Sigma	Cat#11836153001
AflIII	NEB	Cat#R0541
HINDIII-HF	NEB	Cat#R3104
AsiSI	NEB	Cat#R0630
XbaI	NEB	Cat#R0145
Critical commercial assays		
Neon Transfection System 10 μL Kit	ThermoFisher	Cat#MPK1096
DNAeasy Blood and Tissue Kit	Qiagen	Cat#69504
Gel Purification Kit	Qiagen	Cat#28704
PCR Purification Kit	Qiagen	Cat#28104
Quikchange II XL Site-Directed Mutagenesis Kit	Agilent	Cat#200521

(*Continued on next page*)

Continued

REAGENT or RESOURCE	SOURCE	IDENTIFIER
Lipofectamine 3000 transfection kit	ThermoFisher	Cat#L3000008
BluePhos Microwell Substrate Kit, KPL	VWR	Cat#95059-224
ExpiFectamine 293 transfection kit	ThermoFisher	Cat#A14635
**Deposited data**		
Hapmap Project Genotype Data	Frazer et al.[35]	HapMap r28; https://ftp.ncbi.nlm.nih.gov/hapmap/
1000 Genomes Phase 3 Genotype Data	1000 Genomes Project Consortium et al.[37]	v.20130520; https://ftp.1000genomes.ebi.ac.uk/vol1/ftp/
Y. pestis Hi-HOST GWAS summary statistics	This paper	Duke Research Data Repository: https://doi.org/10.7924/r43n2d008
BioBank Japan Chronic HCV summary statistics	Sakaue et al.[76]	BioBank Japan PheWeb: https://pheweb.jp/pheno/CHC
Raw Image and sequencing data from this paper	This paper (Mendeley)	Mendeley Data: https://doi.org/10.17632/hs467zj2cw.1
**Experimental models: Cell lines**		
Human Lymphoblastoid Cell Lines (LCLs)	Coriell Institute	See Table S1 for all LCL individual identifiers
HeLa Cells	Duke Cell Culture Facility, originally from ATCC	ATCC CCL-2
LCL HG02678 KO cells	This paper	N/A
**Oligonucleotides**		
See Table S2 for LCL KO gRNA and primers	Synthego	N/A
See Table S3 for list of GeneBlocks	This paper (IDT)	N/A
See Table S4 for primers	This paper (IDT)	N/A
**Recombinant DNA**		
p67GFP3.1	Pujol & Bliska[97]	N/A
pCMV6	Simon Gregory, PhD	DCK582
pCMV6 *FCRL3*	Origene	Cat#RC214467
pCMV-Sport6 *CD31*	Timothy Wilson, PhD	DCK1056
pFLAG-CMV-3 *FCRL1*	Wilson et al.[47]	DCK1050
pFLAG-CMV-3 *FCRL3*	Wilson et al.[47]	DCK1051
pFLAG-CMV-3 *FCRL4*	Wilson et al.[47]	DCK1052
pFLAG-CMV-3 *FCRL5*	Wilson et al.[47]	DCK1053
pFLAG-CMV-3 *FCRL6*	Wilson et al.[47]	DCK1054
pCMV6 *SYK*	Origene	Cat#RC200413
pCMV6 $FCRL3^{-IGLD1}$	This paper	DCK1196
pCMV6 $FCRL3^{-IGLD2}$	This paper	DCK1197
pCMV6 $FCRL3^{-IGLD3}$	This paper	DCK1198
pFLAG-CMV-3 *FCRL1+FCRL3* ^{IGLD1}	This paper	DCK1230
pCMV6 $FCRL3^{Y65\circ}F$	This paper	DCK1169
pCMV6 $FCRL3^{Y662F}$	This paper	DCK1170
pCMV6 $FCRL3^{Y65\circ}F^{+Y662F}$	This paper	DCK1173
pCMV6 $FCRL3^{Y692F}$	This paper	DCK1171
pCMV6 $FCRL3^{Y722F}$	This paper	DCK1172
pCMV6 $FCRL3^{N721S}$	This paper	DCK1124
pD649-Hasp-COMP5AP CD31	Wojtowicz et al.[74]	Addgene #157481
pD649-Hasp-COMP5AP FCRL5	Wojtowicz et al.[74]	Addgene #157554
**Software and algorithms**		
GraphPad Prism 10	GraphPad Software	www.graphpad.com
R 4.4.0	R Core Team	www.r-project.org

(*Continued on next page*)

Continued

REAGENT or RESOURCE	SOURCE	IDENTIFIER
BioRender	BioRender	www.biorender.com
"locuszoomr" R package	Lewis et al.[38]	https://cran.r-project.org/web/packages/locuszoomr/vignettes/locuszoomr.html
"coloc" R package	Giambartolomei et al.[77]	https://cran.r-project.org/web/packages/coloc/vignettes/a01_intro.html
PLINK 1.9	Chang et al.[98]	www.cog-genomics.org/plink/
ImageJ 1.54g	Schneider et al.[99]	https://imagej.net/ij/
"rptR" R package	Stoffel et al.[100]	https://rdrr.io/cran/rptR/
Code Sample: Generating QQ Plots in R	University of Michigan Center for Statistical Genetics, Matthew Flickinger	https://genome.sph.umich.edu/wiki/Code_Sample:_Generating_QQ_Plots_in_R
"fastman" R package	Paria et al.[101]	https://github.com/kaustubhad/fastman
ICE CRISPR Analysis Tool	Synthego/EditCo	https://www.synthego.com/products/bioinformatics/analysis

EXPERIMENTAL MODEL AND STUDY PARTICIPANT DETAILS

Human cell culture

961 male and female LCLs were purchased from Coriell and cultured for eight days in RPMI 1640 media supplemented with 10% heat-inactivated fetal bovine serum (FBS), 2 mM glutamine, 100 U/mL penicillin-G, and 100 mg/mL streptomycin prior to assays. Cells were passaged at 150,000 cells/ml in 20mL total volume for three days after an initial 1-day rest period. LCLs were counted with a Guava Easycyte Plus flow cytometer (Millipore).

HeLa cells were obtained from Duke University Cell Culture Facility and cultured in DMEM supplemented with 10% fetal bovine serum, 100 U/mL penicillin-G, and 100 mg/mL streptomycin. After 75–90% confluency was reached, cells were washed with PBS and lifted with 0.05% trypsin EDTA before neutralizing with FBS. HeLas were passaged at a 1:5 dilution in 10mL total volume for three days.

PBMCs were purchased from Lonza (CC-2704) and were thawed and recovered 2–12 h in RPMI 1640 media supplemented with 10% heat-inactivated FBS, 2 mM glutamine, 100 U/mL penicillin-G, and 100 mg/m streptomycin. 24TL312076 was obtained from a 28 year old Caucasian female with A-blood type and 24TL249158 was obtained from a 40 year old Caucasian male with A+ blood type. No further information was provided by Lonza.

Bacterial cell culture

Y. pestis (KIM6+ and KIM5) and *Y. pseudotuberculosis* (IP32935) were tagged with an inducible GFP plasmid (p67GFP3.1 from[97]). Bacteria were grown 17 h at 26°C at 250 rpm in Bacto^{tm} Heart Infusion Broth (HIB)(BD, Cat#: 238400) and ampicillin (100 μg/mL) then subcultured by a 1:33 dilution and grown for 2 h 40 min at 37°C.

METHOD DETAILS

Human cell infection assays

Prior to infection, LCLs or PBMCs were washed once with RPMI (no additives) and then plated out in RPMI +0.03% BSA at 100,000 cells/100 μL in a 96-well or 500,000 cells/500 μL in a 24-well non-tissue-culture-treated plate. *Yersinia* was added for 1 h at a multiplicity of infection (MOI) of 30 after centrifugation at 100 x g for 5 min, followed by addition of gentamicin (50 μg/mL) for 1h 60 μL of the infected culture was then split into 140 μL of antibiotic-free media +10% FBS to dilute gentamicin (15 μg/mL). 2 hpi, IPTG (1.4 mM) was added to turn on GFP expression for 120 min prior to 4 h timepoints. 150 μL of cells were stained with 7-AAD (7-aminoactinomycin D; Enzo Life Sciences) and green and red fluorescence of 7000 cells was measured on a Guava Easycyte Plus flow cytometer (Millipore).

In HeLa cells, the same protocol was used, replacing RPMI and RPMI +0.03% BSA with DMEM (no additives) and plating either 12,500/100 μL in a TC-treated 96-well plate or 30,000/200 μL in a chamber well slide. Additionally, the media was replaced with DMEM media +10% FBS, 15 μg/mL gentamicin, and IPTG (1.4 mM) for 120 min following 1 h with high gentamicin.

Cas9-RNP based editing of LCLs

To assemble RNP complexes, Synthego sgRNAs stocks for CD46 (a control transmembrane protein found on all nucleated cells) and FCRL3 at 30 μM and Cas9 at 20 μM were prepared (Table S2). In PCR tubes, 30 pmol of sgRNA and 10 pmol of Cas9 per guide were

mixed and resuspension Buffer R was added to reach 7 μL total per reaction. While the RNP complexes incubated for 10–15 min at room temperature, 500,000 LCLs/reaction were centrifuged at 200 x g for 5 min, washed with PBS, and then suspended in Resuspension Buffer R at a concentration of 500,000 cells/5 μL. 5 μL of the cell suspension was added to each 7 μL of RNP complex mix (either CD46+FCRL3 or just CD46). Using the 10 μL Neon Transfection Kit (ThermoFisher), each reaction was pulsed at 1350 mV for 30 ms. The cells were then transferred into 200 μL of antibiotic free RPMI media +10% FBS to rest for 48 h. After cells reached >2 million, they were stained with PE CD46 (TRA-2-10) flow antibody (BioLegend) in accordance with manufacturers instructions and the CD46 negative population was separated by fluorescence-activated cell sorting. Using Synthego suggested primers (Table S2), regions of interest were sequenced and analyzed using the ICE CRISPR Analysis Tool (EditCo) to ensure >65% of reads contained a frameshift causing a protein knockout of FCRL3. Pooled knockouts A and B for each condition ($CD31^{-/-}$ and $FCRL3^{-/-}$;$CD31^{-/-}$) were used for infections.

HeLa overexpression transfection

Plasmids were transfected using Lipofectamine 3000 per manufacturer's instructions. 12,500/100 μL or 30,000/200 μL HeLa cells were plated on a TC-treated 96-well dish or in a chambered coverslip respectively in DMEM +10% FBS and 1% pen/strep. After 24 h, the media was replaced with DMEM +10% FBS without antibiotics after washing. After 1h, 100 ng DNA, 0.2 μL of P3000 reagent, and 0.2 μL of Lipofectamine 3000 reagent were mixed with 10μL optiMEM and added to each 100 μL well or 2x for 200 μL wells.

HeLa microscopy

Cells were washed with PBS and treated with 4% PFA at 1 or 4 h post infection for 15 min. Each well was then washed three times with PBS. 150 μL of PBS with either 1% saponin +5% normal donkey serum (block/permeabilization solution) or only 5% normal donkey serum (block only, for non-permeabilized cells) was filtered and applied to the cells for 30 min. Primary antibody was applied at the indicated dilutions overnight at 4°C: 9e10 MYC 1:8 (Developmental Studies Hybridoma Bank), NR-31024 Polyclonal Anti-*Yersinia pestis* F1-Antigen 1:20 (BEI Resources), H4A3-s LAMP1 1:50 (Developmental Studies Hybridoma Bank) in block or block/permeabilization solution. The cells were washed three times with PBS and Alexa-fluor conjugated secondary antibodies (ThermoFisher) were added for 1 h at a concentration of 1:1000. DAPI was added to the cells in a concentration of 2.5 μM for 5 min. The cells were washed three times with PBS and all liquid and the plastic guard was removed. Flouromount-G (Invitrogen) was added to each sample and a coverslip was added. The coverslip was left to dry for 24 h before imaging on a Crest X-light V2 spinning disk confocal system (CrestOptics) with a Zeiss Observer Z1 inverted microscope using a 63x water objective or on a EVOS M5000 Microscope with 40x air objective. Final images were adjusted, cropped, and scale bars were added using ImageJ.[99]

Quantification of Syk and FCRL3 clustering by microscopy

Slides were prepared as described above at 1 hpi, using bacteria that have been induced with IPTG during subculture in HIB prior to infection. Images were taken at 40x on an EVOS M5000 Microscope and ~50 infected cells were counted for each condition. The counter was blinded to the conditions until after completion of counting.

Construction of FCRL3 mutants

Deletion of Ig-like domains from FCRL3 were made using gene blocks and cloning methods. Briefly, WT pCMV6-FCRL3 plasmid (RC214467 from OriGene) and 3 gene blocks (Table S3), each containing two of the first three Ig-like domains (Q96P31, nucleotides 61–294 for Ig-like domain 1, 295–546 for Ig-like domain 2, and 574–810 for Ig-like domain 3) were cleaved with AsiSI and XbaI. Restriction-enzyme-digested WT FCRL3 plasmid lacking the first three Ig-like domains was resolved by gel electrophoresis and subsequent purification (Qiagen Gel Purification Kit). The restriction-enzyme-digested gene blocks were also purified (Qiagen PCR Purification Kit). Each digested gene block was ligated into the digested FCRL3 plasmid. The mutant FCRL3 plasmids were then transformed into E. coli (*NEB 5-alpha Competent E. coli*), and successful genetic manipulation was confirmed with sequencing. Similarly, a gene block containing the Ig-like domain 1 in FCRL3 (nucleotides 61–294, see Table S3) was inserted into FCRL1 by restriction using AflIII and HindIII and insertion of the gene block by ligation.

To create point mutants in pCMV6-FCRL3, Lightning QuikChange kit (Agilent) was utilized according to manufacturer's instructions. Primers were created with Agilent's The QuikChange Primer Design Program (Table S4). Mutations were validated by Sanger sequencing.

Purification of FCRL5 extracellular domain and *Y. pestis* binding assay

For the extracellular domain of FCRL5, pD649-HAsp-FCRL5-COMP5AP-AviTag-9xHis (Plasmid #157554 from Addgene[74]) was used. CD31 was again used as the control (pD649-HAsp-CD31-COMP5AP-AviTag-9xHis, Plasmid #157481 from Addgene[74]). These plasmids were transfected into Expi293F cells using the ExpiFectamine 293 transfection kit. Cells were split to 2.5–3$\times 10^6$ viable cells/mL. After 24 h, 20 mL of cells at high density (>5$\times 10^6$ viable cells/mL) were split to a final density of 3$\times 10^6$ viable cells/mL. For each transfection condition, 20000 ng DNA in 1000 μL of Opti-MEM I Reduced Serum Medium were mixed with 54 μL of ExpiFectamine 293 Reagent in 1000 μL of Opti-MEM I and incubated at room temperature for 10–15 min,[74] then added to the seeded cells. 18–22 h

post transfection, 120 μL of ExpiFectamine293 Transfection Enhancer 1 and 1.2 mL of ExpiFectamine 293 Transfection Enhancer 2 were added to the transfected cells. Conditioned media was collected 4 days post transfection.

For purification of His-tagged proteins in conditioned media, 12 mL of conditioned media was added to 10mg of Dynabeads magnetic beads (ThermoFisher). After a 2 h incubation on a roller at 4°C, beads were washed four times with binding/washing buffer (50 mM SodiumPhosphate, pH 8.0, 300 mM NaCl, 0.01% Tween-20). Beads were then incubated for 5 min in 200 μL of elution buffer (300 mM Imidazole, 50 mM Sodium phosphate pH 8.0, 300 mM NaCl, 0.01% Tween-20). Purified supernatant was collected after the beads were applied to the magnet for 2 min.

For detection of direct binding, 3x10^7 *Y. pestis* were incubated with 0.3% BSA in 60μL PBS pH 7.4 (1X) for 15–20 min at 4°C. After which, ~1.8 μg of purified FCRL5 or CD31 control in 30 μL were added for a 90 μL reaction volume. After 30 min at 4°C, *Y. pestis* was spun down (5000 x g for 5 min), washed 2 × 5 min with 100 mM Sodium Phosphate, pH 8.0, 600 mM NaCl, 0.02% Tween-20, and then lysed by boiling in 30 μL PBS pH7.4 (1X) 1x SDS-PAGE loading buffer for 5 min followed by sonication for 2 × 10 s (Qsonica sonicator Q55, 1/8″ Probe 15–20% amplitude). Inputs and bound fractions were resolved on a 4–20% gradient Mini-PROTEAN gel (Bio-Rad) and quantified by western blot probed with Avi Tag (1D11D10) monoclonal antibody (Thermo A01738-40, 1:1000 dilution) with quantification of bands using the western analysis function in LiCor Odyssey Image Studio Ver 4.0 to calculate the percentage of purified protein bound. Similar amounts of purified FCRL5 and CD31 were used in binding reactions based on quantification of purified proteins by western blot probed with anti-AviTag. In four binding experiments using two protein preparations, the relative amount of CD31 vs. FCRL5 used was 1.2 (+/−0.7).

Western blot

Cell lysates were harvested from 100,000 cells using 30μL of TBS +1% Octyl β-D-glucopyranoside (Sigma) and mini-cOmplete protease inhibitor table (Sigma; 1/4th tablet added to 1.5mL of lysis buffer). Tubes were rocked at 4°C for 30 min before being centrifuged at 10,000 g for 5 min. Supernatant was added to 6x SDS loading buffer + βME and then boiled for 10 min. Samples were resolved on a 4–20% gradient Mini-PROTEAN gel (Bio-Rad) for 20 min at 80V then 40 min at 120V. Using the T77 semidry system (Amersham Biosciences (VWR)), proteins were transferred to a PVDF membrane using 60 mAmps. Blot was rinsed in PBS for 10 min and then placed in LICOR Odyssey Blocking Buffer for 1 h at room temperature. Primary antibody was placed in blocking buffer +0.2% Tween overnight at 4°C. E7 beta Tubulin antibody (Developmental Studies Hybridoma Bank) was added to western blots at a 1:100 dilution for 1 h at room temperature to confirm equal loading of samples. The membrane was washed four times for 5 min each in PBS +0.2% Tween-20 and IRDye secondary antibody (IRDye 800CW Goat anti-Rabbit, IRDye 680CW Goat anti-mouse IgG) (LiCor) was added 1:20,000 for 1 h. The membrane was again washed four times for 5 min and then washed one more time in PBS before being imaged on the Licor Odyssey imager with Image Studio Ver 4.0.

Flow cytometry of PBMCs

Following infection, PBMCs were spun down at 300 g for 5 min to pellet and washed with PBS+2% Fetal Bovine Serum. The cells were then incubated in PBS+2% Fetal Bovine Serum for 15 min at room temperature. Cells were stained with the manufacturer's suggested concentration of PE anti-human CD3 (UCHT1)(BD Biosciences, 561808), and PE/CY5 CD19 (HIB19)(eBioscience, 15-0199-71) for 30 min at 4°C in PBS+2% FBS. Cells were washed with PBS and flow cytometry was performed after resuspending in 200 μL of PBS+2% FBS.

QUANTIFICATION AND STATISTICAL ANALYSIS

Genome-wide association

For each of 961 LCLs, three independent experiments were performed on sequential passages, and the mean was taken as the final invasion (4 h % GFP+ cells) phenotype (see Table S1). Repeatability was calculated under a linear mixed model using the R package "*rptR*",[100] with 1000 bootstraps (for standard error and CI) and 1000 permutations (for permutation-based *p*-value).

Genotype imputation and genome-wide association analysis were performed as described in our previously published work.[27,29] Briefly, Hi-HOST was performed on 434 LCLs from HapMap project (CEU, YRI, CHB, JPT)[34] and 527 LCLs from 1000 Genomes Project (ESN, GWD, IBS, KHV).[102] Genotypes for the HapMap LCLs were obtained from HapMap r28 while genotypes for the 1000 Genomes Project LCLs were extracted from the 1000 Genomes Project Phase 3 genotypes (v.20130520). Both the HapMap and 1000 Genomes genotypes were imputed against the 1000 Genomes Project Phase 3 reference (GRCh37/hg19). Imputed genotypes were combined based on shared SNPs. A MAF filter of <0.01 was applied, resulting in a total of 15,213,612 filtered SNPs.

Using the above genotypes, GWAS was conducted in PLINK v1.9[98] using the QFAM-parents approach with adaptive permutation and a maximum of 10^9 permutations. This approach performs linear regression to test for association while employing permutation of within- and between-family components separately to control for population stratification.[42] The human genome reference assembly (GRCH37/hg19) was used for all analysis.

Phenotype- and SNP-based heritability analysis

Two different methods were applied to estimate heritability, as described for other Hi-HOST traits.[29] Briefly, the parent-offspring (PO) regression method estimated additive heritability using only phenotypic values and family relationships. Here, the slope is used as an

estimation of heritability and was calculated at 0.1883. Secondarily, a genotype-based heritability estimate was performed using the GCTA GREML method.[103] Here, SNPs with MAF of <0.05 were excluded to construct the genetic relationship matrix (GRM). Zaitlen's GREML method, which enables estimating GRM using both related and unrelated individuals, was used because of the family trio design used in Hi-HOST. The SNP-based h^2 was 0.1881.

Colocalization analysis of Hi-HOST *Y. pestis* phenotype and BioBank Japan chronic hepatitis C GWAS

The R package "*coloc*" which is based on Giambartolomei et al.'s colocalization analysis,[77] was used to determine if GWAS signals are due to a shared causal SNP. This method calculates the posterior probabilities that two traits are not associated in the locus of interest (PP0), only one trait is associated in the locus (PP1 and PP2), both traits are associated at the locus but with different, independent causal variants (PP3), or both traits are associated with a single causal variant in the locus (PP4). For the Hi-HOST and BioBank Japan GWAS summary statistics, we filtered SNPs within a 500 kilobase (kb) window centered on rs2282284. The "coloc.abf" function was executed using the default prior parameters ($p1 = 1 \times 10^{-4}$, $p2 = 1 \times 10^{-4}$, and $p12 = 1 \times 10^{-5}$). PP4 between 0.700 and 0.900 indicated that the traits are likely to share a single causal variant. The PP4/PP3 indicated the intensity of the colocalization signal with values > 5.00 further supporting colocalization.

Descriptive statistics and visualization

Descriptive statistics were performed with GraphPad Prism 10 (GraphPad Software, US) and with R Studio.[104] QQ plots were plotted using an adapted version of "Code Sample: Generating QQ Plots in R" (University of Michigan Center for Statistical Genetics, Matthew Flickinger) and Manhattan plots were created using "*fastman*".[101] Regional Manhattan plot were made using "*locuszoomr*".[38,105] The size of each study or number of replicates, along with the statistical tests performed can be found in Figure Legends. All statistical tests comparing groups used two-sided *p*-values. All individual dots in figures represent measurements taken from distinct samples.

Cell Genomics, Volume *5*

Supplemental information

Human genetic variation reveals FCRL3 is a lymphocyte receptor for *Yersinia pestis*

Rachel M. Keener, Sam Shi, Trisha Dalapati, Liuyang Wang, Nicolás M. Reinoso-Vizcaino, Micah A. Luftig, Samuel I. Miller, Timothy J. Wilson, and Dennis C. Ko

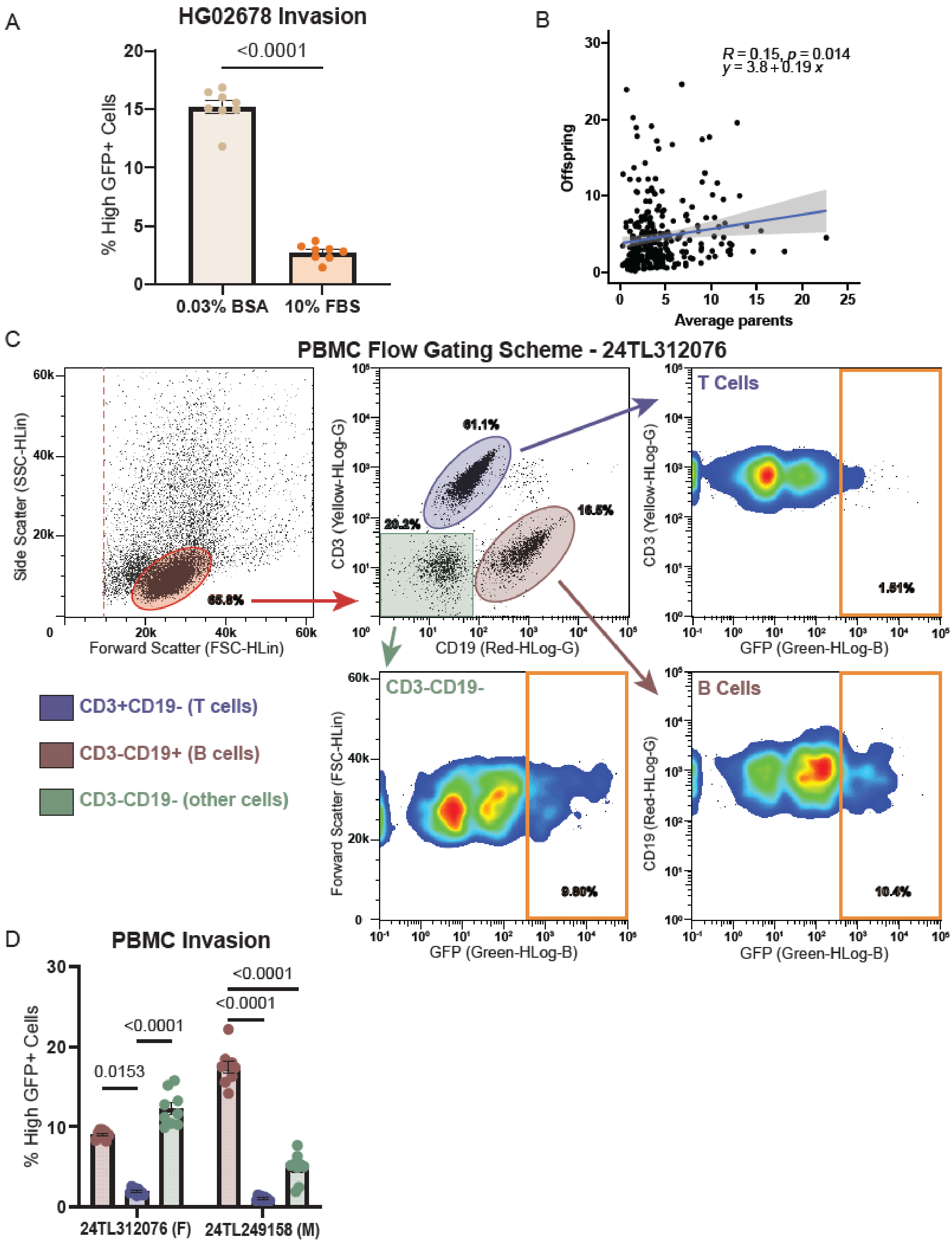

A
HG02678 Invasion
<0.0001
% High GFP+ Cells
0.03% BSA
10% FBS
B
R = 0.15, p = 0.014
y = 3.8 + 0.19 x
Offspring
Average parents
C
PBMC Flow Gating Scheme - 24TL312076
Side Scatter (SSC-HLin)
Forward Scatter (FSC-HLin)
CD3 (Yellow-HLog-G)
CD19 (Red-HLog-G)
T Cells
GFP (Green-HLog-B)
CD3-CD19-
B Cells
CD3+CD19- (T cells)
CD3-CD19+ (B cells)
CD3-CD19- (other cells)
D
PBMC Invasion
% High GFP+ Cells
0.0153
<0.0001
<0.0001
<0.0001
24TL312076 (F)
24TL249158 (M)

Figure S1. *Y. pestis* invasion into LCLs and PBMCs, related to figure 1. (**A**) Flow cytometric measurement of *Y. pestis* (KIM6+ +p67GFP3.1) invasion of LCLs at 4 hpi in RPMI media with either 10% FBS or 0.03% BSA after gentamicin protection assay. 2 experiments are grand mean normalized with 8 total replicates plotted for each condition. An unpaired t-test was performed to determine significance. (**B**) Parent-offspring linear regression of *Y. pestis* invasion into LCLs gives a correlation coefficient of 0.15, a slope and estimated heritability of 0.1883 and a p-value = 0.014. (**C**) Flow cytometry gating scheme for *Y. pestis* infected PBMCs. After infecting cells in serum free RPMI +0.03% BSA for 1 hour, a gentamicin protection assay was performed. Cells were then stained with CD19 (B cell marker) and CD3 (T cell marker) before counting 15000 cells on a Guava Easycyte HT flow cytometer using the InCyte program. First, viable cells are gated in the red gate in the top left dot plot. T cells are then defined in the blue gate, B cells in brown, and CD3-CD19- cells in green in the top middle dot plot. Each population is then separated, and invasion (GFP^{HIGH}) is measured in three density plots. (**D**) Bar graph quantifying flow cytometry of PBMCs demonstrates B cell preference for invasion. Dots represent 3 replicates in each of 3 experiments for PBMCs from each donor. P-value determined by two-way ANOVA with Sidak's multiple comparison test.

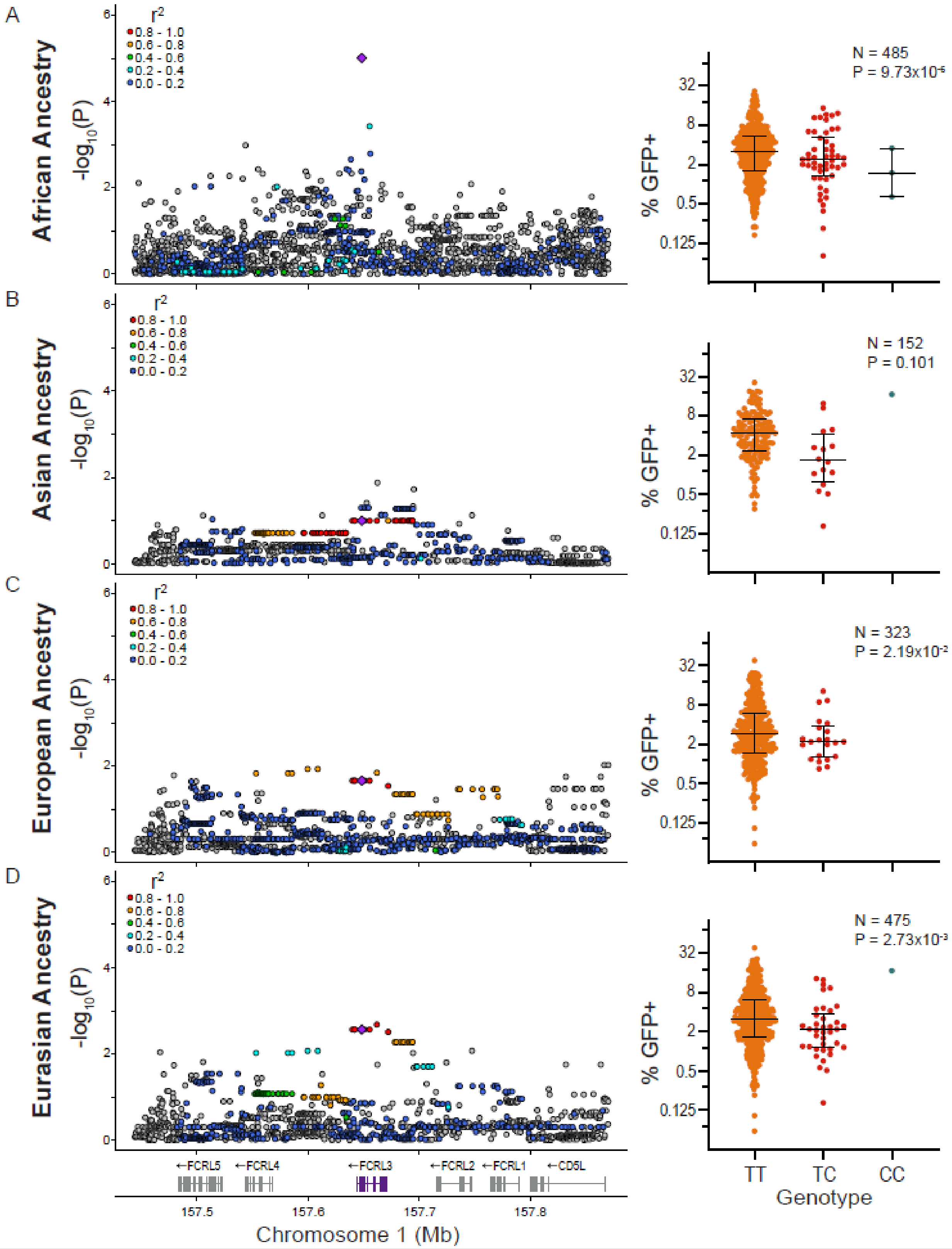

A
B
C
D
African Ancestry
Asian Ancestry
European Ancestry
Eurasian Ancestry
-log10(P)
r2
0.8 - 1.0
0.6 - 0.8
0.4 - 0.6
0.2 - 0.4
0.0 - 0.2
N = 485
P = 9.73x10-6
N = 152
P = 0.101
N = 323
P = 2.19x10-2
N = 475
P = 2.73x10-3
% GFP+
32
8
2
0.5
0.125
←FCRL5
←FCRL4
←FCRL3
←FCRL2
←FCRL1
←CD5L
157.5
157.6
157.7
157.8
Chromosome 1 (Mb)
TT
TC
CC
Genotype

Figure S2. **Locus zoom and genotypic mean plots of LCLs stratified by ancestry, related to figure 1.** Local Manhattan plots and genotypic mean plots stratified by African (GWD, YRI, ESN), Asian (JPT, CHB, KHV), European (CEU, IBS), or EurAsian (JPT, CHB, KHV, CEU, IBS) ancestries. Plot shows the *FCRL3* locus flanked on either side by 200kb. A purple diamond denotes rs2282284 and LD with SNPs in the locus is shown by red >= 0.8, orange = 0.6-0.8, green = 0.4-0.6, light blue = 0.2-0.4, dark blue < 0.2, and grey has no LD data. LD was determined by all populations within the stratified group. -$\log_{10}(p)$ values at the rs2282284 locus was plotted and phenotypes were plotted by genotype. All populations demonstrate the C allele of rs2282284 is associated with decreased invasion.

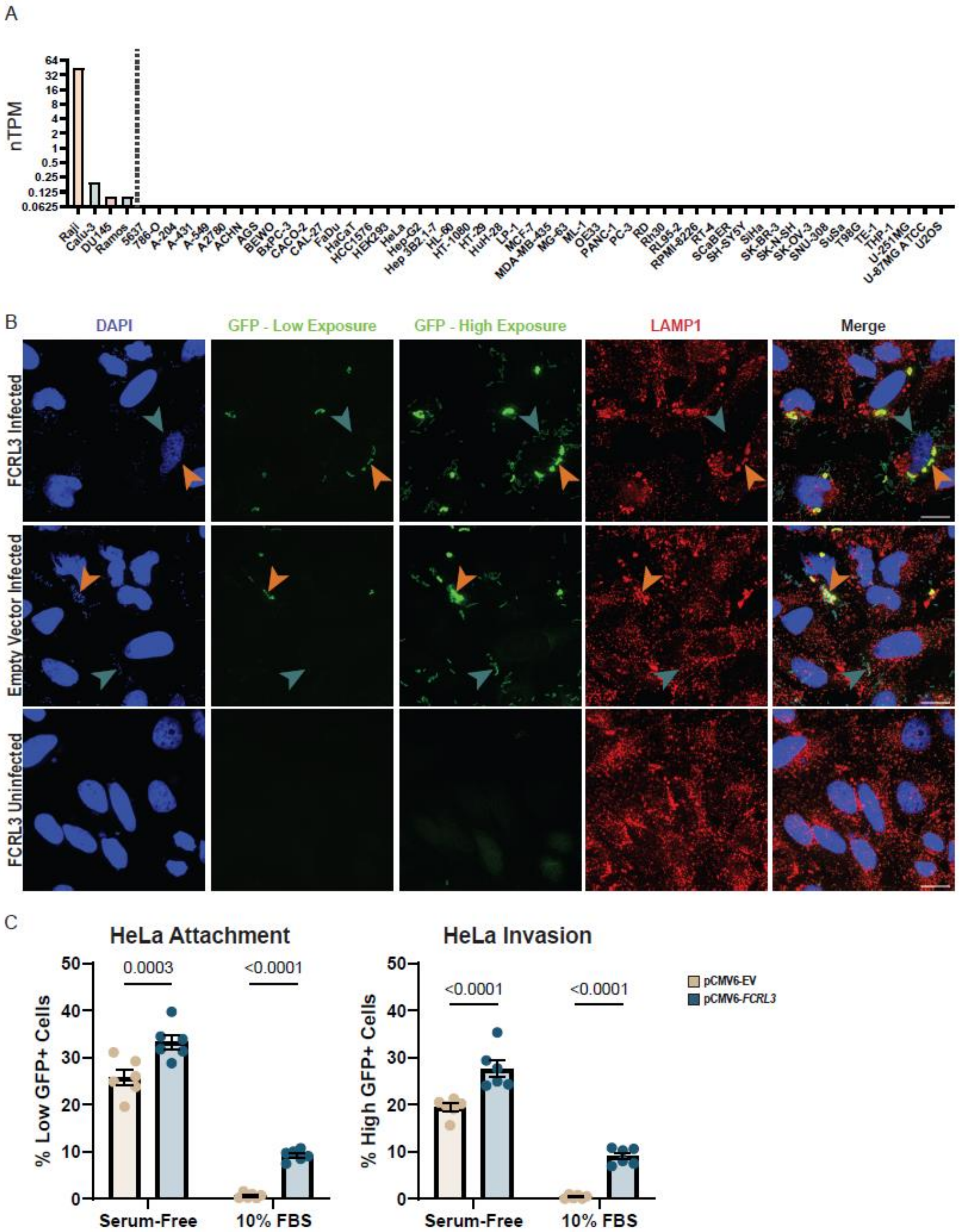
A
nTPM
64
32
16
8
4
2
1
0.5
0.25
0.125
0.0625
Raji
Calu-3
DU145
Ramos
5637
786-O
A-204
A-431
A-549
A2780
ACHN
AGS
BEWO
BxPC-3
CACO-2
CAL-27
FaDu
HaCaT
HCC1576
HEK293
HeLa
Hep-G2
Hep 3B2.1-7
HL-60
HT-1080
HT-29
HuH-28
LP-1
MCF-7
MDA-MB-435
MG-63
ML-1
OE33
PANC-1
PC-3
RD
Rh30
RL95-2
RPMI-8226
RT-4
SCaBER
SH-SY5Y
SiHa
SK-BR-3
SK-N-SH
SK-OV-3
SNU-308
SuSa
T98G
TE-1
THP-1
U-251MG
U-87MG ATCC
U2OS
B
DAPI
GFP - Low Exposure
GFP - High Exposure
LAMP1
Merge
FCRL3 Infected
Empty Vector Infected
FCRL3 Uninfected
C
HeLa Attachment
% Low GFP+ Cells
0.0003
<0.0001
Serum-Free
10% FBS
HeLa Invasion
% High GFP+ Cells
<0.0001
<0.0001
Serum-Free
10% FBS
50
40
30
20
10
0
pCMV6-EV
pCMV6-FCRL3

Figure S3. No FCRL3 protein is detected in HeLa cells and intracellular *Y. pestis* is in LAMP+ vesicles at 4 hpi, related to figure 2. (**A**) Bar graphs of expression in common cell lines from the Human Protein Atlas[S1] show expression only in lymphoma, Calu-3, and DU145 cell lines and no expression in HeLa. (**B**) GFP^{high} cells have intracellular *Y. pestis* within LAMP1+ vacuoles. HeLa cells transfected with empty vector or *FCRL3* plasmid were infected with KIM6+ +p67GFP3.1 *Y. pestis* for 1 hr, treated with gentamicin for 1 hr, and induced with IPTG for 2 hr prior to fixation with 4% paraformaldehyde. After incubating for 30 minutes in block/perm, DNA was stained with 2.5μM DAPI, LAMP1 was stained red with a 1:20 dilution of H4A3-s mouse LAMP1 antibody (Developmental Studies Hybridoma Bank), and bacteria are shown in green at high exposure to include the GFP^{low} population. GFP^{low} bacteria are indicated with a teal arrowhead and GFP^{high} bacteria with an orange arrowhead. Images were taken on a Zeiss Observer Z1 inverted microscope with a 63x water objective. A 20μm scale bar is located on the merged image. ImageJ was used to adjust and crop images and to add scale bars. (**C**) Serum reduces FCRL3-independent attachment and invasion in HeLa cells overexpressing pCMV6 empty vector or FCRL3. HeLa cells were transfected with empty vector or FCRL3 plasmid and assayed for attachment and invasion + or – 10% Fetal Bovine Serum by flow cytometry. Three biological replicates in each of two experiments were plotted and a two-way ANOVA was performed with Uncorrected Fisher's LSD multiple comparison test to determine the P-value. Experiments were normalized by grand mean.

Table S2. sgRNAs and primers for CRISPR mutagenesis and validation related to figure 2 and STAR methods.

GENE	GUIDE 1	GUIDE 2	GUIDE 3	FWD primer	REV primer
**CD46**	GAGAAACAUGUCCAUAUAUA	AACUCGUAAGUCCCAUUUGC	UUGCUCCUUAGAGGAAAUAA	TGCCTGGGTGAATATGAATCTT	TGTCAGAAACAGCAAGTAGTTTTG
**FCRL3**	AAUUUCCAGGCUCUGUAAUU	GAUACCAAUAUGUGUCUCCC	CUGUGGACCAUGGAGGAUUG	TCTGCCTAGGATCCCTGCAT	ACCCTGGTCCTGACTGGA

Table S3. GeneBlocks used in IG-like domain deletion and insertion cloning, related to figure 5 and STAR methods.

FCRL3 Ig-Like Domain	Parental Plasmid	Sequence
D1 Deletion	pCMV6 FCRL3	AGCTATGCGATCGCCATGCTTCTGTGGCTGCTGCTGCTGATCCTGACTCCT<br>GGAAGAGAACAATCAGGGGTGGCCCCTGACTGGCTGATCCTGCAGGCTTT<br>ACATCCTGTCTTTGAAGGAGACAATGTCATTCTGAGATGTCAGGGGAAAG<br>ACAACAAAAACACTCATCAAAAGGTTTACTACAAGGATGGAAAACAGCTTC<br>CTAATAGTTATAATTTAGAGAAGATCACAGTGAATTCAGTCTCCAGGGATA<br>ATAGCAAATATCATTGTACTGCTTATAGGAAGTTTTACATACTTGACATTGA<br>AGTAACTTCAAAACCCCTAAATATCCAAGTTCAAGAGCTGTTTCTACATCCT<br>GTGCTGAGAGCCAGCTCTTCCACGCCCATAGAGGGGAGTCCCATGACCCT<br>GACCTGTGAGACCCAGCTCTCTCCACAGAGGCCAGATGTCCAGCTGCAATT<br>CTCCCTCTTCAGAGATAGCCAGACCCTCGGATTGGGCTGGAGCAGGTCCCC<br>CAGACTCCAGATCCCTGCCATGTGGACTGAAGACTCAGGGTCTTACTGGTG<br>TGAGGTGGAGACAGTGACTCACAGCATCAAAAAAAGGAGCCTGAGATCTC<br>AGATACGTGTACAGAGAGTCCCTGTGTCTAATGTGAATCTAGATCAGCC
D2 Deletion	pCMV6 FCRL3	AGCTATGCGATCGCCATGCTTCTGTGGCTGCTGCTGCTGATCCTGACTCCT<br>GGAAGAGAACAATCAGGGGTGGCCCCAAAAGCTGTACTTCTCCTCAATCC<br>TCCATGGTCCACAGCCTTCAAAGGAGAAAAAGTGGCTCTCATATGCAGCA<br>GCATATCACATTCCCTAGCCCAGGGAGACACATATTGGTATCACGATGAGA<br>AGTTGTTGAAAATAAAACATGACAAGATCCAAATTACAGAGCCTGGAAATT<br>ACCAATGTAAGACCCGAGGATCCTCCCTCAGTGATGCCGTGCATGTGGAAT<br>TTTCAATCCAAGTTCAAGAGCTGTTTCTACATCCTGTGCTGAGAGCCAGCTC<br>TTCCACGCCCATAGAGGGGAGTCCCATGACCCTGACCTGTGAGACCCAGCT<br>CTCTCCACAGAGGCCAGATGTCCAGCTGCAATTCTCCCTCTTCAGAGATAG<br>CCAGACCCTCGGATTGGGCTGGAGCAGGTCCCCCAGACTCCAGATCCCTG<br>CCATGTGGACTGAAGACTCAGGGTCTTACTGGTGTGAGGTGGAGACAGTG<br>ACTCACAGCATCAAAAAAAGGAGCCTGAGATCTCAGATACGTGTACAGAG<br>AGTCCCTGTGTCTAATGTGAATCTAGATCAGCC
D3 Deletion	pCMV6 FCRL3	AGCTATGCGATCGCCATGCTTCTGTGGCTGCTGCTGCTGATCCTGACTCCT<br>GGAAGAGAACAATCAGGGGTGGCCCCAAAAGCTGTACTTCTCCTCAATCC<br>TCCATGGTCCACAGCCTTCAAAGGAGAAAAAGTGGCTCTCATATGCAGCA<br>GCATATCACATTCCCTAGCCCAGGGAGACACATATTGGTATCACGATGAGA<br>AGTTGTTGAAAATAAAACATGACAAGATCCAAATTACAGAGCCTGGAAATT<br>ACCAATGTAAGACCCGAGGATCCTCCCTCAGTGATGCCGTGCATGTGGAAT<br>TTTCACCTGACTGGCTGATCCTGCAGGCTTTACATCCTGTCTTTGAAGGAGA<br>CAATGTCATTCTGAGATGTCAGGGGAAAGACAACAAAAACACTCATCAAA<br>AGGTTTACTACAAGGATGGAAAACAGCTTCCTAATAGTTATAATTTAGAGA<br>AGATCACAGTGAATTCAGTCTCCAGGGATAATAGCAAATATCATTGTACTG<br>CTTATAGGAAGTTTTACATACTTGACATTGAAGTAACTTCAAAACCCCTAAA<br>TATCCAAGTTCAAGAGCTGTTTCTACATAAAAGGAGCCTGAGATCTCAGAT<br>ACGTGTACAGAGAGTCCCTGTGTCTAATGTGAATCTAGATCAGCC

D1 Insertion	pFLAG-CMV-3 FCRL1	AGCTATAAGCTTGCGGCCCCAAAAGCTGTACTTCTCCTCAATCCTCCATGGTCCACAGCCTTCAAAGGAGAAAAAGTGGCTCTCATATGCAGCAGCATATCACATTCCCTAGCCCAGGGAGACACATATTGGTATCACGATGAGAAGTTGTTGAAAATAAAACATGACAAGATCCAAATTACAGAGCCTGGAAATTACCAATGTAAGACCCGAGGATCCTCCCTCAGTGATGCCGTGCATGTGGAATTTTCAGCGGAGCTGTTTTTGATAGCCAGCCCCTCCCATCCCACAGAGGGGAGCCCAGTGACCCTGACGTGTTCAGCC

Table S4. Oligonucleotides for Quikchange mutagenesis of FCRL3, related to figure 5 and STAR Methods.

mutation	Forward	reverse
N721S	catgaagaagatgatgaagaaagctatgagaatgtaccacgtgta	tacacgtggtacattctcatagctttcttcatcatcttcttcatg
Y650F	ggagctggagccaatgttcagcaatgtaaatcctg	caggatttacattgctgaacattggctccagctcc
Y662F	ctggagatagcaacccgattttttcccagatctg	cagatctgggaaaaaatcgggttgctatctccag
Y692F	gaggaacttacagtcctcttttcagaactgaagaagaca	tgtcttcttcagttctgaaaagaggactgtaagttcctc
Y722F	cccatgaagaagatgatgaagaaaactttgagaatgtaccac	gtggtacattctcaaagttttcttcatcatcttcttcatggg

Supplemental references

S1. Uhlen, M., Fagerberg, L., Hallstrom, B.M., Lindskog, C., Oksvold, P., Mardinoglu, A., Sivertsson, A., Kampf, C., Sjostedt, E., Asplund, A., et al. (2015). Proteomics. Tissue-based map of the human proteome. Science *347*, 1260419. 10.1126/science.1260419.
